# Comparative Analysis of *Mycoplasma gallisepticum* vlhA Promoters

**DOI:** 10.3389/fgene.2018.00569

**Published:** 2018-11-21

**Authors:** Mikhail Orlov, Irina Garanina, Gleb Y. Fisunov, Anatoly Sorokin

**Affiliations:** ^1^Institute of Cell Biophysics, Russian Academy of Sciences, Pushchino, Russia; ^2^Federal Research and Clinical Center of Physical-Chemical Medicine, Federal Medical-Biological Agency, Moscow, Russia

**Keywords:** *Mycoplasma gallisepticum*, promoter, transcription regulation, DNA physics, vlhA

## Abstract

*Mycoplasma gallisepticum* is an intracellular parasite affecting respiratory tract of poultry that belongs to class Mollicutes. *M. gallisepticum* features numerous variable lipoprotein hemagglutinin genes (vlhA) that play a role in immune escape. The vlhA promoters have a set of distinct properties in comparison to promoters of the other genes. The vlhA promoters carry a variable GAA repeats region at approximately 40 nts upstream of transcription start site. The promoters have been considered active only in the presence of exactly 12 GAA repeats. The mechanisms of vlhA expression regulation and GAA number variation are not described. Here we tried to understand these mechanisms using different computational methods. We conducted a comparative analysis among several *M. gallisepticum* strains. Nucleotide sequences analysis showed the presence of highly conserved regions flanking repeated trinucleotides that are not linked to GAA number variation. VlhA genes with 12 GAA repeats and their orthologs in 12 *M. gallisepticum* strains are more conserved than other vlhA genes and have narrower GAA number distribution. We conducted comparative analysis of physicochemical profiles of *M. gallisepticum* vlhA and sigma-70 promoters. Stress-induced duplex destabilization (SIDD) profiles showed that sigma-70 group is characterized by the common to prokaryotic promoters sharp maxima while vlhA promoters are hardly destabilized with the region between GAA repeats and transcription start site having zero opening probability. Electrostatic potential profiles of vlhA promoters indicate the presence of the distinct patterns that appear to govern initial stages of specific DNA-protein recognition. Open state dynamics profiles of vlhA demonstrate the pattern that might facilitate transcription bubble formation. Obtained data could be the basis for experimental identification of mechanisms of phase variation in *M. gallisepticum*.

## Introduction

Mycoplasmas are genome-reduced bacteria without a cell wall and with a parasitic lifestyle. Mycoplasmas parasitize diverse animal and plant species and humans. Like other intracellular parasites, they need to adapt to the host’s immune system. One of main mechanisms Mycoplasmas employ is changing the repertoire of surface lipoproteins (phase variation) (Rosengarten and Wise, 1990). Other pathogenic bacteria, including *Haemophilus*, *Chlamydia*, and *Streptococcus* species, also use phase variation to escape of host defense mechanisms (Noormohammadi, 2007). Phase variation in Mycoplasmas can occur spontaneously or due to an immune attack, it is important for persistence and survival of Mycoplasmas in a host (Markham et al., 1998; Glew et al., 2000; Ma et al., 2015; Czurda et al., 2017; Chopra-Dewasthaly et al., 2017). Numerous mechanisms of phase variation are described for Mycoplasmas (Citti et al., 2010). Usually, the mechanisms of variation are species-specific and occur in one species or closely related Mycoplasmas. They include DNA slippage, site-specific recombination, reciprocal recombination, and gene conversion (Citti et al., 2010). However, the phase variation system of *Mycoplasma gallisepticum* is unique, and has not been described so far. Therefore, studying phase variation genes can reveal novel mechanisms of gene expression regulation in bacteria.

*Mycoplasma gallisepticum* is a major bacterial pathogen inducing widespread respiratory disease in poultry and wild birds, which leads to significant economic losses throughout the world (Bencina, 2002). Phase variation of *M. gallisepticum* includes the switching on variable lipoprotein and hemagglutinin (vlhA) gene expression (Markham et al., 1992). The exact function of vlhA proteins is still unknown. They involve in haemagglutination (Bencina, 2002; Noormohammadi, 2007), based on data obtained on avian Mycoplasmas it can be assumed that vlhA proteins participate in host cell adhesion and invasion (May et al., 2014; Matyushkina et al., 2016; Hegde et al., 2018). VlhA genes are organized into 3–5 cassettes, uniting ten genes per cassette (Baseggio et al., 1996). The promoter structure of these genes is significantly different from the promoters of the other *M. gallisepticum* genes. VlhA genes lack conserved sigma-70 promoter sequence and often have GTG start codon (Markham et al., 1994). They are proposed to employ an alternative sigma factor binding GCGAAAAT sequence (Fisunov et al., 2016). Long regions of GAA repeats are located upstream of vlhA genes (Markham et al., 1994). In general, the GAA repeats can be considered as short-sequence repeats (SSRs). SSRs were found in all eukaryotic and many prokaryotic genomes (Mrázek et al., 2007; Avvaru et al., 2017). In bacteria, SSRs were identified in genes coding for bacterial virulence factors including lipopolysaccharide-modifying enzymes or adhesins (Mrázek, 2006; Wei et al., 2015). So, SSRs provide genetic and, therefore, phenotypic variability. Changes in number of repeated units and/or in the repeat unit itself may arise from recombination processes or polymerase errors including slipped-strand mispairing (SSM), either solely or in combination with DNA repair deficiencies (van Belkum et al., 1998; Rocha, 2003; Torres-Cruz and van der Woude, 2003).

First experiments showed that *M. gallisepticum* express only one vlhA family member at a time and expression depends on the presence of exactly 12 GAA trinucleotide repeats upstream of the gene (Glew et al., 1995, 1998; Liu et al., 2002). Recently it was shown that expression of the gene preceded by 12 GAA exceeds the other vlhA genes, but the other genes with a different number of repeats are also expressed and some of them are expressed at a high level (Matyushkina et al., 2016; Pflaum et al., 2016; Butenko et al., 2017). *In vivo* experiments showed the non-stochastic character of vlhA switching during infection, vlhA expression pattern changes during infection progression and differs between strains (Pflaum et al., 2016, 2018). So, vlhA expression is determined by GAA repeats, but probably the additional expression control mechanisms exist. An interesting question here is how the cell defines what promoter needs to be activated. One explanation here is the existence of hemagglutinin activator protein (HAP) recognizing 12-GAA repeats (Liu et al., 2002).

Another question is the mechanism of GAA repeat variation in *M. gallisepticum*. It would be interesting to find out how many repeats changes at a time, whether the change depends on the number of repeats of a given gene, or on the sequences surrounding the GAA repeats and their physicochemical properties. In the present study we used computational methods to analyze genomes of several *M. gallisepticum* strains and shed light to the mechanism of phase variation and vlhA expression control. For this purpose, we used comparative bioinformatics analysis of sequences of vlhA promoters and genes. We assumed that a nonstandard structure of vlhA promoters may be related to the physicochemical properties of their sequences, using computational methods we predicted these properties on the DNA of vlhA promoters and compared them with the corresponding properties of experimentally obtained sigma-70 promoters of *M. gallisepticum S6*.

## Materials and Methods

### Bioinformatics Analysis of (GAA)n and vlhA Genes

We used 12 complete genomes of *M. gallisepticum* strains isolated from chickens and house finches of various levels of virulence available for download in June 2018 in the GenBank database (Papazisi et al., 2003; Szczepanek et al., 2010; Fisunov et al., 2011; Tulman et al., 2012; Fleming-Davies et al., 2018). List of the genomes and their characteristics (size, GC content, and number of genes) are provided in Supplementary Table S1. We obtained sequences of vlhA promoters of all 12 strains to study GAA number variation. For comparison of physicochemical properties, we retrieved sequences of sigma-70 promoters of S6 strain. The exact coordinates of the transcription start sites of *M. gallisepticum* S6 were obtained from our published work there 5′-end enriched RNA-seq sequencing was conducted (Mazin et al., 2014).

The GAA repeats were defined as 4–27 non-interspaced trinucleotides repeated in a row. A smaller number of the repeats appeared to be non-specific; no 28 or more repeats were detected. We proposed that for the possible GAA recognizing protein the length of GAA tract should be more important than the substitutions in one repeat inside the (GAA)n. So, we considered units with substitutions inside the (GAA)n as intact units and shortened the (GAA)n to the units with at least one substitution if it was at the end of the (GAA)n. We did not detect GAA tracts containing more than two damaged GAA inside the tract. For sequencing retrieval and GAA counting we used Python 2.7 custom script.

To analyze GAA number variation we classified vlhA genes into orthologous groups. Not all vlhA have clear annotation, most are annotated as hypothetical proteins. Since we are interested only in vlhA under the control of (GAA)n containing promoters, to find all vlhA genes we first mapped GAA repeats and then found corresponding vlhA genes. Several times we observed short GAA repeat in coding regions of other genes or GAA that not connected with vlhA, this cases we corrected manually. ProteinOrtho program (version V5.16) was used to computing orthologous vlhA proteins (Lechner et al., 2011). Parameters identity =70% and minimum coverage of best blast alignments =50% were used. Fisher exact test was performed using fisher.test() function in R with two.sided alternative hypothesis.

To reconstruct the phylogenetic tree of vlhA genes for Figure 3 we obtained consensus sequences of orthologous clusters applying Biopython command dumb_consensus() to orthologous group alignments (Cock et al., 2009). VlhA proteins and their consensus sequences we aligned by T-coffee program implemented in JalView software (version 2.10.5) with default parameters (Waterhouse et al., 2009; Di Tommaso et al., 2011). Phylogenetic tree of consensus sequences was constructed by Phylogeny.fr tool where the method of maximum-likelihood is implemented (Dereeper et al., 2008). The histogram of GAA number and distributions were constructed in R.

### Analysis of (GAA)n Flanks

For analysis of (GAA)n flanking regions, we extracted 50 nucleotide sequences upstream and downstream of the (GAA)n. We aligned upstream and downstream flanks independently by T-Coffee program implemented in JalView software (version 2.10.5) with default parameters (Waterhouse et al., 2009; Di Tommaso et al., 2011) and merged corresponding aligned flanks using Biopython Python 2.7 library (Cock et al., 2009). See flanks alignment in Supplementary Materials. WebLogo was used for sequence logos construction (Crooks et al., 2004).

To compare (GAA)n flanking sequences between 12-GAA and the other vlhA genes we used a non-linear algorithm of dimension reduction t-SNE (t-Distributed Stochastic Neighbor Embedding). t-SNE allows a visualization a high-dimensional data to see high-dimensional objects in two- or three-dimensional space. t-SNE visualizes the data in compact and clear view and has advantages over other dimension reduction methods, like PCA (van der Maaten and Hinton, 2008). Alignment was transformed into the table presenting nucleotides and gaps with numbers, columns correspond to positions in alignment, rows to individual genes. We employed PCA algorithm with default parameters and t-SNE algorithm with perplexity parameter 30 implemented in sklearn Python 2.7 library (Pedregosa et al., 2011).

### Calculation of Physicochemical Properties of Promoters

Stress-induced duplex destabilization (SIDD) is a theoretical method developed to analyze denaturation in superhelical DNA of a specified sequence (Benham, 1990). SIDD profile analysis predicts the DNA positions where the DNA duplex becomes susceptible to separation when under superhelical stress (Benham, 1990). SIDD calculation was carried out as implemented by its authors (Zhabinskaya et al., 2015). The conformational and thermodynamic parameters were derived from the endonuclease digestion experiments on superhelical DNA (Kowalski et al., 1988; Benham, 1992). Theoretical calculations using these parameters were consistent with experimental data (Benham, 1992).

For SIDD calculations 1000 nts-long intervals with transcription start site (TSS) at the center were considered, usage long DNA regions take into account broader genomic context. We filtered nucleotide sequences containing more than one promoter. SIDD profiles were obtained by means of perl script. SIDD calculation was performed using default settings (superhelicity level 0.06, energy threshold 12, and ionic strength 0.01). Temperature value was equal to the average chicken body temperature (314 K). The difference between SIDD profile maximum values was tested by the non-parametric Mann–Whitney *U* test implemented in R using wilcox.test() function with parameter paired =FALSE.

Distribution of electrostatic potential is DNA duplex feature that contributes to the initial stages of DNA–protein interactions (Jones et al., 2003). The DNA characteristic profiles were obtained using method suitable for genome-wide application (Polozov et al., 1999). The approach is based on Coulomb formula and allows to analyze electrostatic profiles of promoters within the electrostatic map of a whole genome DNA. It is widely used in studies concerning electrostatic patterns of bacterial and phage promoters (Polozov et al., 1999; Kamzolova et al., 2005, 2006, 2009; Sorokin et al., 2006; Osypov et al., 2010). Finally, DNA open states dynamical properties, including their activation energy (E0) and size (d). These are believed to affect transcription bubble formation and introduce additional to the encoded by steady-state DNA properties information. The used model equation was derived from the sine-Gordon equation by adding two additional terms which more accurately take into account heterogeneous nature of the DNA sequence. The profiles were shown to be in agreement with the function of the corresponding DNA regions: promoters are evolving open states with most ease, while terminator are likely to stop the transcription bubble (Grinevich et al., 2015). Therefore, SIDD profiles were obtained by means of perl script, electrostatic profiles was calculated using the algorithm implemented in R, and the dynamical properties of DNA open states were obtained using the algorithm implemented in Matlab 9.2.

## Results

### VlhA Promoters Share Conserved GAA-Flanking Sequences Irrespective of GAA Units Number

**FIGURE 1:**
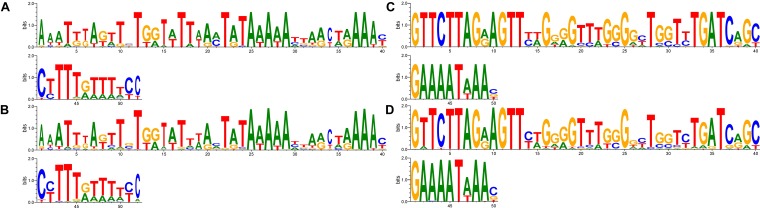
The motif of (GAA)n flanking sequences in vlhA promoters. Logos show identity of motifs for promoters with different GAA number. Sequences 50 bp length were aligned by T-coffee program, gaps included in the alignment. Logo constructed by WebLogo 3.6.0; **(A,B)** logos for upstream flanks, **(C,D)** logos for downstream flanks; **(A,C)** logos of 22 sequences of 12-GAA promoters; **(B,D)** logos of 344 non-12-GAA promoters.

Comparative analysis of GAA repeats number for vlhA genes of different strains was conducted to identify possible patterns of variation. All vlhA genes from 12 strains were clustered into orthologous groups according to the sequence similarity. Previous studies revealed that activation of vlhA transcription occurs if 12 GAA repeats are present within the promoter. Flanking regions of the GAA repeats were also found to be essential for vlhA expression (Liu et al., 2000). Here we analyzed conservation of GAA flanks among different *M. gallisepticum* strains and vlhA orthologous groups to identify the mechanism of vlhA expression activation. For each vlhA gene sequences upstream and downstream of (GAA)n were obtained. Totally 368 promoters were taken into analysis. GAA tracts were defined as repeat regions containing 4 or more GAA trinucleotides without substitutions at the ends of the (GAA)n. The logos build demonstrate conserved sequences both upstream and downstream of GAA repeats (Figure 1). The conservation level varies among positions of the motifs. We searched for similar sequences in nucleotide collection at NCBI blast by blastn program and did not find any matches in other species. So, these sequences show no sequence homology with sequenced genomes and appear to be identified in *M. gallisepticum* genome only. The sequences comprise neither repetitive sequences nor palindromes that often are present in regulatory motifs.

**FIGURE 2:**
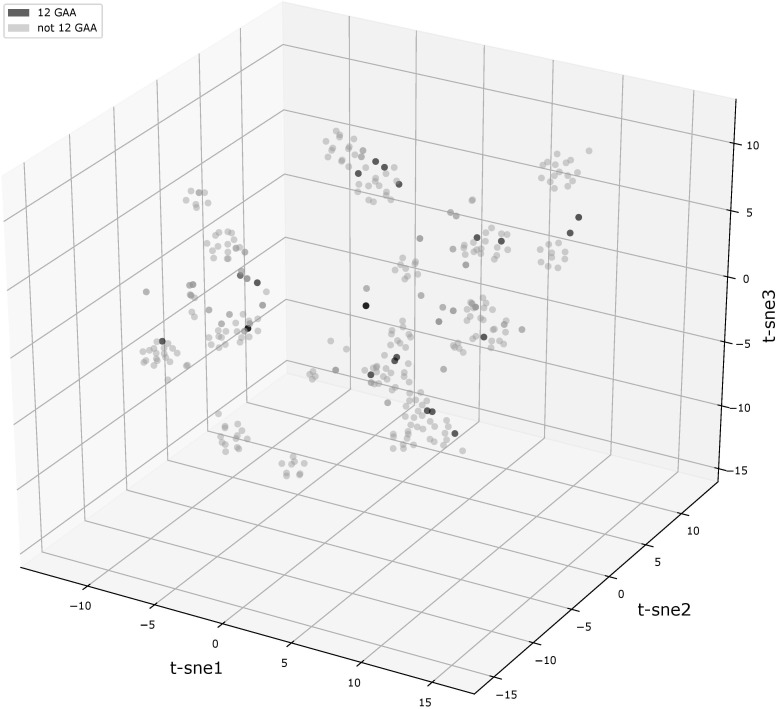
t-SNE analysis plot of (GAA)n flanking motifs. Points represent individual vlhA genes of all analyzed strains, the analysis made on concatenated left and right (GAA)n flanking sequences. Black points show 12-GAA promoters. In analysis was used t-SNE algorithm implemented in sklearn Python library with the parameter of perplexity =30.

We compared flanking sequences of 12-GAA tracts with other vlhA promoters. First, we looked over logos of 12-GAA and non-12-GAA flanks (Figure 1). No traceable distinction was found between the two groups. To more precise comparison we visualized sequences in three-dimensional space using t-SNE method (Figure 2). This method shows sequences similarity as a distance in two- or three-dimensional space. No clustering of promoters with 12 GAA was identified by t-SNE and by similar method PCA (Supplementary Figure S1). So, analysis of GAA flanking regions revealed conserved positions around GAA tract and did not show correlations between 12-GAA units in (GAA)n and sequence of (GAA)n flanks.

**FIGURE 3:**
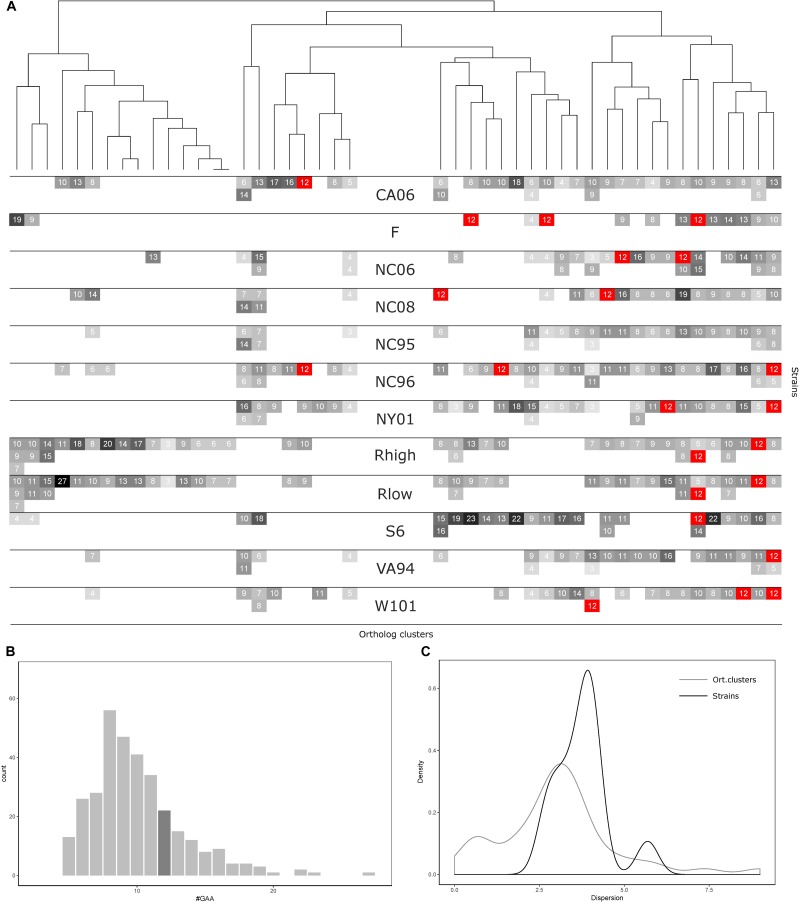
GAA repeats number statistics for 12 *Mycoplasma gallisepticum* strains and vlhA orthologous clusters. **(A)** Heatmap showing number of GAA repeats for each vlhA promoter. The number of repeats is indicated by colors, 12-GAA repeats are shown with red. One strain corresponds to three rows (three is the maximum numbers of vlhA paralogs observed for a strain). Names of the strains are shown in the heatmap center. Orthologous clusters correspond to columns. The tree was constructed by Phylogeny.fr software based on T-coffee protein alignment of consensus sequences of orthologous groups using the maximum-likelihood method for phylogeny reconstruction. **(B)** Histogram of the number of GAA repeats. The dark gray bar shows 12-GAA promoters. **(C)** Distribution of dispersion of GAA repeats number among strains and orthologous clusters.

To consider in more detail the flanking sequences, we constructed their alignments and phylogenetic trees for genes belonging to the same orthologous groups. In the article we describe two representative examples of trees (Figure 3) and the alignments of flanks of orthologous groups (Supplementary Materials). The identity level between vlhA proteins of these two orthologous groups is higher than 90% for all protein pairs. The first tree represents the tree of the merged flanks of (GAA)n for the orthologous cluster containing 4 genes with 12-GAA repeats. This is the largest orthologous group, containing proteins represented in all strains. The alignment and tree show that the sequences are conservative within the groups of strains isolated from different species: strains F, S6, Rlow, and Rhigh were obtained from chickens, the remaining strains from house finches. Genomes of finch strains have almost identical genome sequences with a low number of substitutions, but the difference exists (Tulman et al., 2012; Kristensen et al., 2017). Chicken strains are less similar to each other than strains from finches according to data from the ATGC database (Tulman et al., 2012). That is, in this case, one would expect slight differences between the (GAA)n flanks of individual strains, but the sequences for the orthologous group are completely identical within two groups. It is interesting that the flanks and the corresponding genes are located in different vlhA cassettes, the genes from chicken strains are located in the first cassette, and finch genes are located in the third and fourth cassettes. So, the moving to other cassette did not affect sequences of (GAA)n flanks. The orthologous group includes 4 genes with 12-GAA repeats, no differences between them and other genes are noticeable. We observed that the number of repeats within the orthologs cluster varied, while sequences of repeats were conservative. This suggests that the change in the number of GAA repeats does not depend on the sequences flanking them. Figure 3B shows the tree of another orthologous group, which also contains 12-GAA repeat genes. The tree confirms the lack of connection between the number of repeats and the sequence of flanks. These flanking sequences are less conservative among themselves than sequences of the first group. Thus, analysis of trees and alignments of particular orthologous groups showed no connections between (GAA)n number and their flanking sequences.

### Number of GAA Repeats Varies Among Orthologs vlhA and Different Strains of *M. gallisepticum*

**FIGURE 4:**
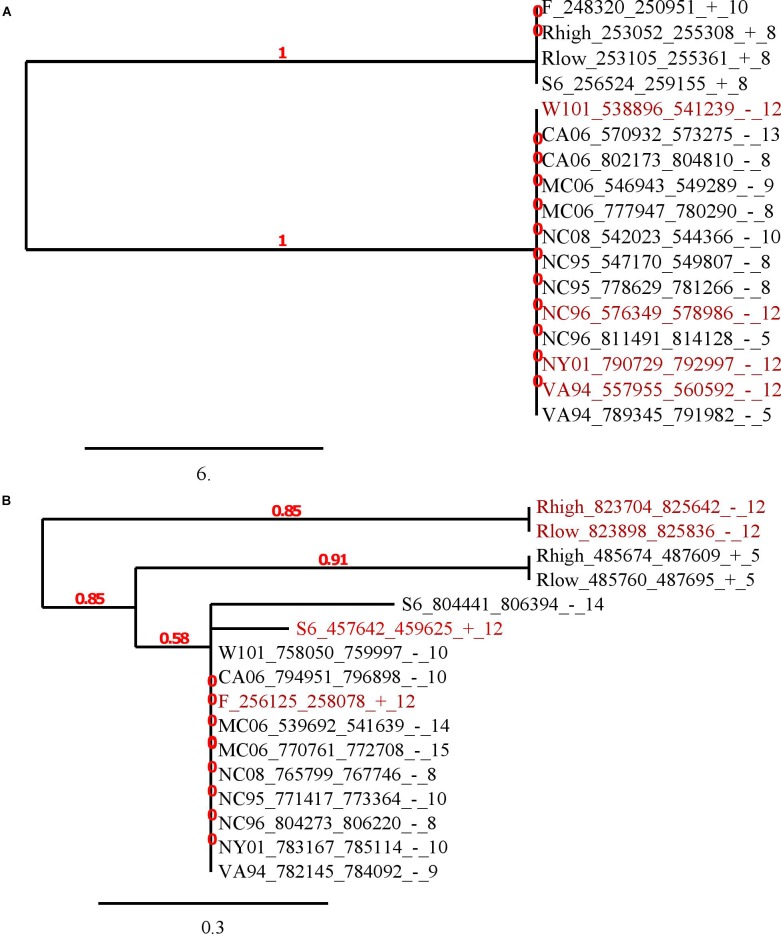
Phylogenetic trees of (GAA)n flanking sequences. The trees were constructed by Phylogeny.fr software based on T-coffee nucleotide alignments of 50 bp flanks. The maximum-likelihood method was applied for phylogeny reconstruction. Labels of sequences consist of strain short name, corresponding vlhA gene position (start, end, strand separated by ‘_’) and GAA units number separated by ‘_’. Red color shows 12-GAA promoters. The scale bar shows 0.02 changes. Red numbers on branches display branch support values. **(A)** The tree of the biggest orthologous group that is depicted in the last column in Figure 3A; **(B)** the tree of another orthologous group, consisting of four 12-GAA genes. The group is depicted in 41 column in Figure 3A.

Comparative analysis of GAA repeats a number of vlhA genes from different strains were conducted to identify possible patterns of GAA number variation. All vlhA genes from 12 strains were clustered into orthologous groups (Figure 4A). The distribution of GAA tract lengths shows that the majority of values reside within a narrow range of 6-12 repeats. We divided vlhA orthologous clusters into two groups: the one containing 12 repeats at least in one strain and the one including the rest. The distribution within 12-GAA containing group is even narrower varying from 8 to 12 repeats. This may indicate that GAA number changes by an increase/decrease of a small number of repeats.

The number of 12-GAA promoters varies across the strains from zero to three per genome. We found the positive correlation between gene conservation level and the presence of 12-GAA repeats within an ortholog cluster. Genes with 12 repeats are more frequently occur in full ortholog clusters comprising to genes that are represented in all strains (Fisher exact test *p*-value =0.0248).

The number of repeats varies within one genome as well as within one orthologous cluster. We analyzed the distribution of GAA repeats number among the strains and orthologs clusters (Figures 4B,C). The data shows that the prevalent GAA repeats number is 8 and frequency decreases as the number of repeats increases. Genes with 12 GAA repeats follow the common trend and have no exceptional frequencies. Comparison of dispersion in repeats number among the strains and ortholog clusters showed that the number of repeats is more conserved within one strain than within one ortholog cluster. The majority of the strains tend to follow this trend, except for S6 strain which exhibited the most versatile repeat number. Certain ortholog clusters are more conserved than others which may indicate differences in VlhA expression among strains. Therefore, analysis of GAA repeats number did not reveal any traceable patterns in the distribution of repeats. We suggest that alike patterns might be established after considering a bigger set of strains.

### VlhA Promoters Have Lowest Opening Probability Under Superhelical Stress (SIDD Profiles) While Non-vlhA Promoters Are Highly Destabilized

**FIGURE 5:**
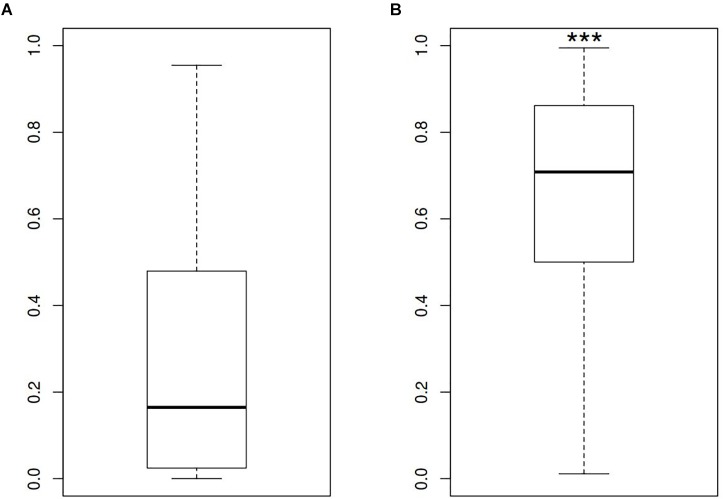
SIDD profile maximum values for **(A)** vlhA promoters of 12 *M. gallisepticum* strains and **(B)** sigma 70 promoters of S6 strain. ^∗∗∗^Mann–Whitney test *p*-value <0.05.

**FIGURE 6:**
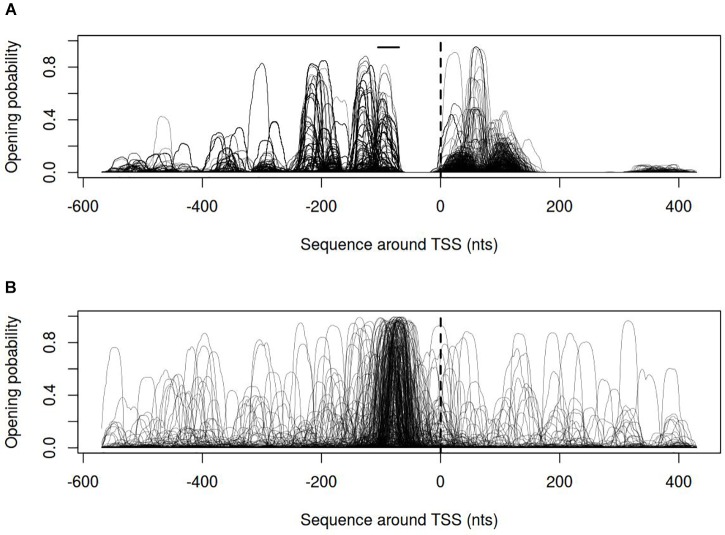
Opening probability (SIDD) profiles for **(A)** vlhA promoters of 12 *M. gallisepticum* strains; **(B)** sigma 70 promoters of S6 strain. Dashed line denotes transcription start site; solid horizontal line – approximate GAA repeats location.

In order to describe the possible role of physicochemical interactions in phase variation of *M. gallisepticum* several DNA properties of promoter regions were obtained in the form of profiles. SIDD as a DNA parameter shows a robust correlation with various regulatory DNA loci including promoters, replication origins, etc. The promoters of E. coli can be classified into SIDD-dependent and SIDD-independent groups according to their SIDD profile, which seems to correlate with their functional specialization (Wang and Benham, 2006). In the present article we analyzed SIDD profiles for vlhA promoters from various *M. gallisepticum* strains as well as, for standard sigma-70 promoters experimentally identified in S6 strain (Mazin et al., 2014). Promoters of both type feature same GC-content of 0.3, which is the average GC-content of *M. gallisepticum* genome. Sigma-70 promoters are substantially more destabilized with the profile maxima located in the vicinity of TSS, while vlhA promoters did not incline to melt under the considered conditions (Figure 5). Peaks of vlhA promoters’ profiles do not overlap TSS region with the sequence adjacent to GAA repeats having zero melting probability. At the same time, the majority of sigma-70 promoters demonstrate sharp maxima in the upstream region [−100; −50] nts (Mann-Whitney test *p*-value <0.05) (Figure 6). The fact to some extent supports the notion that there is no direct correlation between SIDD profiles and GC-content of a DNA segment.

### Dynamical Properties of DNA Open States and Electrostatic Potential Profiles of vlhA Promoters Show Distinct Patterns

**FIGURE 7:**
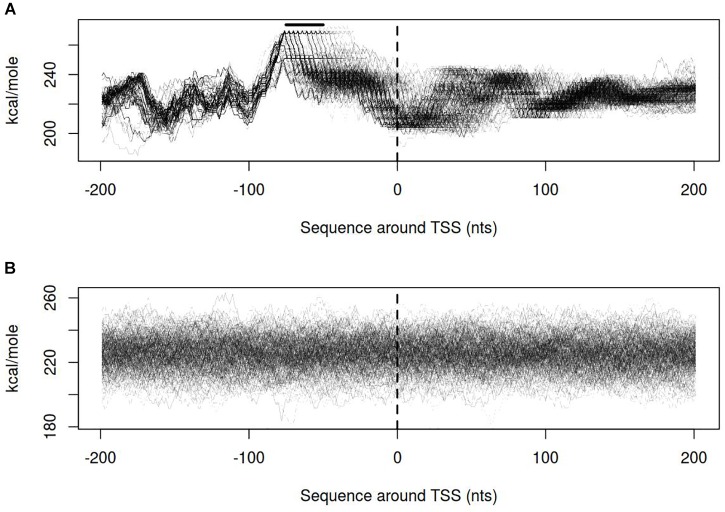
Open states activation energy profiles for **(A)** vlhA promoters of 12 *M. gallisepticum* strains; **(B)** sigma 70 promoters of S6 strain. Dashed line denotes transcription start site; solid horizontal line – approximate GAA repeats location.

**FIGURE 8:**
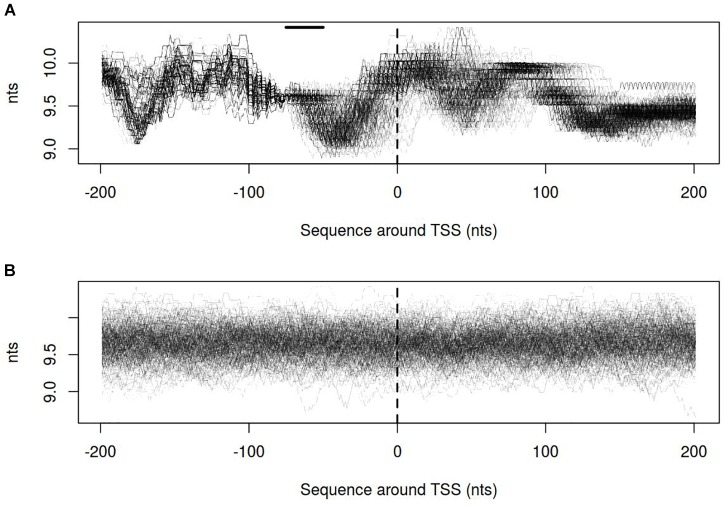
Size of open states profiles for **(A)** vlhA promoters of 12 *M. gallisepticum* strains; **(B)** sigma 70 promoters of S6 strain. Dashed line denotes transcription start site; solid horizontal line – approximate GAA repeats location.

Dynamics of DNA open states was shown to be important for transcription bubble formation (Grinevich et al., 2015). The lower the open states activation energy, the more the DNA duplex is prone to open thus facilitating transcription initiation. Open states activation energy profiles, as well as the size of open states profiles, were calculated for vlhA and sigma-70 promoters. We identified that the transition of vlhA promoters to an open state occurred more efficiently in the region downstream TSS. The activation energy for the promoter group in the interval [-70; 20] nts appeared to have a decreasing slope which starts at the right GAA repeats boundary. It may seem tempting to suggest that the slope facilitate the directed movement of RNA-polymerase along the promoter. At that, no traceable patterns were detected for sigma-70 promoters (Figures 7,8).

**FIGURE 9:**
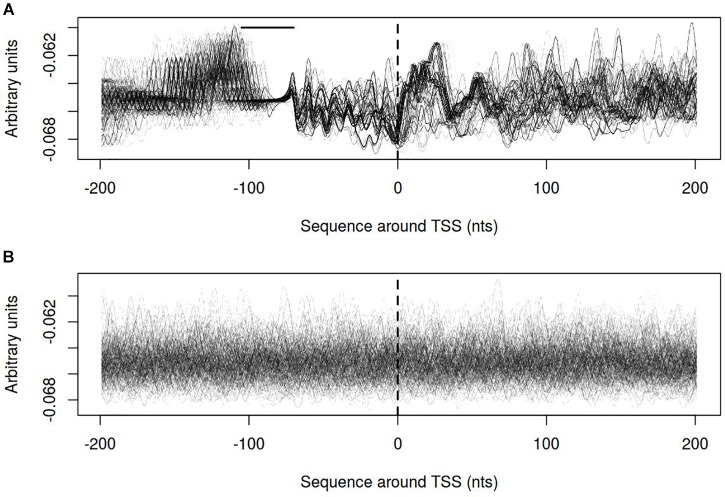
Electrostatic potential (EP) profiles for **(A)** vlhA promoters of 12 *M. gallisepticum* strains; **(B)** sigma 70 promoters of S6 strain. Dashed line denotes transcription start site; solid horizontal line – approximate GAA repeats location.

Distribution of electrostatic potential (EP) around DNA duplex is a physical property that could be recognized by other molecules at a distance and prior to their direct interaction. It appears to be crucial at the initial stages of promoter recognition by RNA-polymerase (Polozov et al., 1999). Promoters of vlhA genes show characteristic EP pattern with the peak at about 30 nt after TSS. Neither visual assessment nor clusterization revealed traceable patterns for sigma-70 promoters profiles (Figure 9).

## Discussion

The promoters of vlhA genes feature a remarkable mechanism of transcriptional regulation. It includes two functional components: transcriptional activation at 12-GAA containing promoters and variation of GAA repeats number. In the article we have analyzed conservation, GAA number distribution, and physicochemical properties of vlhA promoters in *M. gallisepticum*. We proposed that physicochemical properties of promoters including SIDD, DNA open states dynamics, and electrostatic potential could be connected to the vlhA genes expression regulation.

We demonstrated that the GAA repeats in vlhA promoters are flanked by highly conserved sequences with distinct structure. Altogether the regulatory region takes more than 50 nt. Sequences of such length are generally too large for binding a typical bacterial transcription factor (Rodionov, 2007). Regulatory sequences of this length are unique in bacteria. It is possible that *M. gallisepticum* has unique DNA binding proteins with the unknown spatial structure of the DNA binding region that standard annotation programs cannot identify. The hypothesis is supported by the fact that Mycoplasmas have a large number of orphan genes with unknown functions (Tatarinova et al., 2016).

Most of the analyzed strains are isolated from wild birds and are pathogenic for the host. We observed 12-GAA vlhA genes occur more than one time in the genome. Obtained data implies that the presence of a single 12-GAA vlhA gene is not the only possible combination enabling pathogenicity manifestation. Closely related strains Rlow and Rhigh demonstrate similar distributions of 12-GAA genes but have distinct virulence potential (Szczepanek et al., 2010). Vaccine strains F with a low level of pathogenicity have the maximum number of genes with 12-GAA repeats and lacks numerous vlhA genes. One can speculate the inability of proper vlhA switching may result in a decrease of pathogenicity.

We identified that the distribution of GAA number resides within narrow borders of 8–12 repeats only in case orthologous clusters with at least one 12-GAA promoter were considered. We hypothesize that there is a “working range” of GAA repeats within which the number can iterate while having a considerable chance to get back to 12. Promoters that occasionally go out of range are not functional, while they still may remain conserved. The corresponding genes will never be activated again. The orthologous clusters lacking 12-GAA promoters are distributed in considerably fewer strains which corroborates with the idea that they lost function and represent a decaying group of vlhA.

Calculation of physical properties of vlhA promoters and sigma-70 promoters of S6 strain allowed to identify distinct patterns in open states dynamics and electrostatic potential profiles. We hypothesize that the former could facilitate transcription bubble formation thus stimulating processive transcription, while the latter could contribute to the initial stage of DNA-protein recognition. By contrast, SIDD profiles of vlhA promoters are hardly destabilized and have zero opening probability near TSS while sigma-70 promoters have overall high destabilization levels with maxima associated with TSS position. It corroborates with the idea that an alternative sigma-factor rather than sigma-70 is utilized for transcription of vlhA. One can speculate that zero open probability of vlhA promoters under superhelical stress reflects that fact that these loci are wrapped around activator complex, e.g., are at a high local degree of negative supercoiling. At the same time, improper transcription should not be facilitated from vlhA promoters since their −10 boxes show a substantial degree of similarity with those of sigma-70.

## Conclusion

Analysis of promoters of vlhA indicates the presence of conserved sequences upstream and downstream to GAA repeats. Sequences of (GAA)n flanks are not connected with the number of GAA repeats. The distribution of (GAA)n length among the strains of *M. gallisepticum* shows a preferred range within which this number iterates: 6–12 repeats. Distribution of GAA units number varies among strains and orthologous groups. VlhA orthologous groups having at least one 12-GAA gene in the group have a narrower distribution of GAA number with values within the range 8–12 and are more conserved among strains than other orthologous groups. As compared to sigma-70 promoters of *M. gallisepticum* promoters of vlhA feature distinct and characteristic profiles of physical properties including opening probability under superhelical stress, open state activation energy, and electrostatic potential.

## Data Availability

The datasets analyzed and scripts for this study can be found in the https://github.com/FVortex/Orlov_et_al._Frontiers_in_Genetics_Mycoplasma_gallispeticum_script.

## Author Contributions

IG contributed in analysis of genomes, GAA repeats, cauterization, and writing the manuscript. MO contributed in analysis of physicochemical properties and writing the manuscript. GF and AS wrote the manuscript.

## Conflict of Interest Statement

The authors declare that the research was conducted in the absence of any commercial or financial relationships that could be construed as a potential conflict of interest.

## Supplementary Material

TABLE S1 Description of strains used in the study.

TABLE S2 Data on vlhA genes, sequences of genes, their promoters and GAA repeats.
