# Supplementary material for: Comparative Analysis of Mycoplasma gallisepticum vlhA Promoters
Source: Front Genet. 2018 Nov 21;9:569. doi: 10.3389/fgene.2018.00569 (PMC6258824; doi:10.3389/fgene.2018.00569)
Supplement: TABLE S1 — Description of strains used in the study. [file Table_1.DOCX]

***Supplementary Material***

**Comparative analysis of Mycoplasma gallisepticum vlhA promoters**

**M. Orlov, I. Garanina*, Fisunov, A. Sorokin**

*** Correspondence:** I. Garanina: [irinagaranina24@gmail.com](mailto:irinagaranina24@gmail.com)

**1 Supplementary figures**
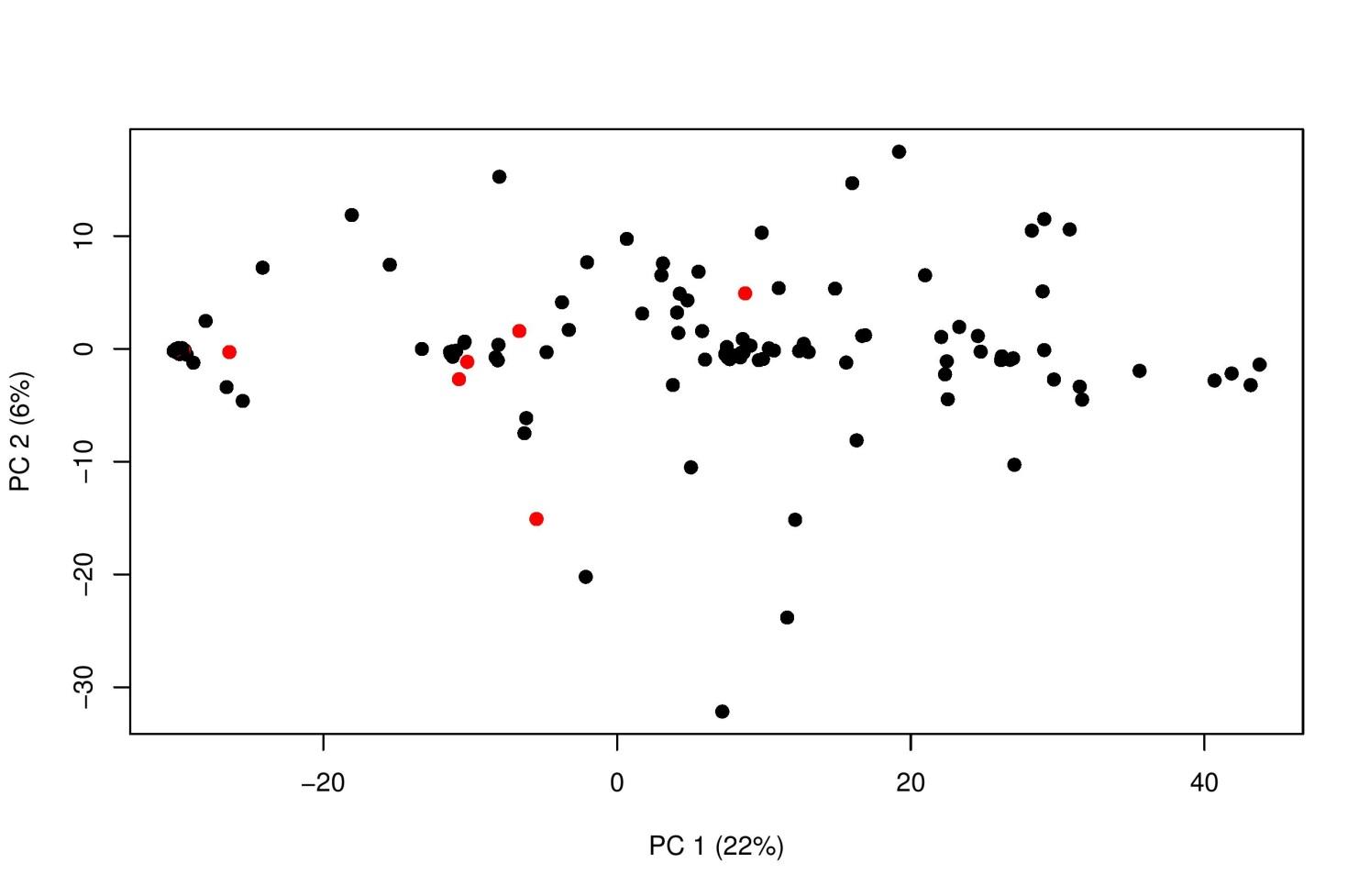


**Supplementary Figure 1.** PCA analysis plot of (GAA)n flanking motifs. Points represent individual VlhA genes of all analyzed strains, the analysis made on concatenated left and right (GAA)n flanking sequences. Red points show 12-GAA promoters. In analysis was used PCA algorithm implemented in sklearn Python library.

# 2 Supplementary Data

**2.1 Alignments of (GAA)n flanking regions**

1. Alignment of 22 upstream flanks of 12-GAA (GAA)n

>8_Rlow_823898_825836_-_12/1-50

---ATGAGTTGTGATGGTTTTAACTATAAAAACTGA-TAAAATCTTTTGTTTTC----------

>9_NC08_770496_772596_-_12/1-50

-----ATTGAGTTGTTGTATTTAATATATAAAATAGCTAAAATCCTTTAAAAATC---------

>5_F_793650_795630_-_12/1-50

---ATAACTTGTGATGGTTTTAACTGTAAAAACTCA-TAAAATCTTTTGTTTTC----------

>3_Rlow_835839_837918_-_12/1-50

------TGTAGATCTGGTTTA-GATATAAATATTTATAGAATTCTTTTATTTTCTAA-------

>15_NC08_268663_270718_+_12/1-50

-----ATTGAGTTGTTGTATTTAATATATAAAATAGCTAAAATCCTTTAAAAATC---------

>17_MC06_544421_546560_-_12/1-50

-----ATTGAGTTGTTGTATTTAATATATAAAATAGCTAAAATCCTTTAAAAATC---------

>0_VA94_557955_560592_-_12/1-50

---AATTGTAGATTTGGTTTT-AATATAAATATCAACAAAA-CCTTTTGTTTTCC---------

>27_NY01_263592_265662_+_12/1-50

--TAAATT-ATTTTTGA-ATTAACTGTAAAAATGAA-TGAAACCTTTTGTTTTCC---------

>0_NY01_790729_792997_-_12/1-50

---AATTGTAGATTTGGTTTT-AATATAAATATCAACAAAA-CCTTTTGTTTTCC---------

>33_NC96_466262_468440_+_12/1-50

-GTAAATT-ATTTCTGA-ATTAACTGTAAAAACGAA-TGAAAGCTTTTGTTTCC----------

>0_W101_538896_541239_-_12/1-50

---AATTGTAGATTTGGTTTT-AATATAAATATCAACAAAA-CCTTTTGTTTTCC---------

>3_Rhigh_835651_837730_-_12/1-50

------TGTAGATCTGGTTTA-GATATAAATATTTATAGAATTCTTTTATTTTCTAA-------

>24_MC06_537255_539352_-_12/1-50

AAAACATT--TCTCTGA-ATTTGCTATAAAAATTAG-TGAAACCTTTGGTTTGC----------

>12_W101_529263_531270_-_12/1-50

---GAATTTAATTGTGGATTA-ACTACAAAAACATATAAAAACCTTTTGTTTTC----------

>8_S6_457642_459625_+_12/1-50

--TAAATT-ATTTCTGA-ATTAACTATAAAAATGAA-TGAAACTTTTTGTTTTCC---------

>14_W101_769847_771884_-_12/1-50

-----ATTGAGTTGTTGTATTTAATATATAAAATAGCTAAAATCCTTTAAAAATC---------

>34_NC96_463816_465964_+_12/1-50

-----ATTGAGTTGTTGTATTTAATATATAAAATAGCTAAAATCCTTTAAAAATC---------

>47_F_258414_260535_+_12/1-50

AAAACATT--TCTCTGA-ATTTGTTATAAAAATTAG-TGAAACCCTTAATTTTC----------

>0_NC96_576349_578986_-_12/1-50

---AATTGTAGATTTGGTTTT-AATATAAATATCAACAAAA-CCTTTTGTTTTCC---------

>8_Rhigh_823704_825642_-_12/1-50

---ATGAGTTGTGATGGTTTTAACTATAAAAACTGA-TAAAATCTTTTGTTTTC----------

>8_F_256125_258078_+_12/1-50

--TAAATT-ATTTCTGA-ATTAACTGTAAAAATGAA-CGAAACCTTTTGTTTTCC---------

>33_CA06_460842_463020_+_12/1-50

-GTAAATT-ATTTCTGA-ATTAACTGTAAAAACGAA-TGAAAGCTTTTGTTTCC----------

## 2. Alignment of 22 downstream flanks of 12-GAA (GAA)n

>8_Rlow_823898_825836_-_12/1-50

GTTCTTAGGAGTTCTGGGGTTTGGGGCTGGTTTGATCAGTGAAAATTAAG

>9_NC08_770496_772596_-_12/1-50

GTTCTTAGAAGTTTAGGAGTTAGCGGATGCTCTGATCGGCGAAAATAAAT

>5_F_793650_795630_-_12/1-50

GTTCTTAGAAGTTTTGGGGTTTGGGAATCCTGTGATCAGCGAAAATTAAG

>3_Rlow_835839_837918_-_12/1-50

GTTCTTAGAAGTTTAGAAGCTAGGGGTTGGTTTGATCAGCGAAAATAAAC

>15_NC08_268663_270718_+_12/1-50

GTTCTTAGAAGTTAGGGGAGTTTGGTCTGGCTTGATCTGCGAAAATAAAC

>17_MC06_544421_546560_-_12/1-50

GTTCTTAGAAGTTTAGGAGTTAGCGGATGCTCTGATCAGCGAAAATAAAC

>0_VA94_557955_560592_-_12/1-50

GTTCTTAGGAGTTCTGTGGTCTGGGGTTGGTTTGATCAGCGAAAATAAAC

>27_NY01_263592_265662_+_12/1-50

GTTCTTAGGAGTTCTGGGGTTTGGGGCTGGTTTGATCAGTGAAAATTAAG

>0_NY01_790729_792997_-_12/1-50

GTTCTTAGGAGTTCTGTGGTCTGGGGTTGGTTTGATCAGCGAAAATAAAC

>33_NC96_466262_468440_+_12/1-50

---CTTAGAAGTTTAGAGGTTTGGGGCTCGTCTGATCGACGAAAATAAAC

>0_W101_538896_541239_-_12/1-50

GTTCTTAGGAGTTCTGTGGTCTGGGGTTGGTTTGATCAGCGAAAATAAAC

>3_Rhigh_835651_837730_-_12/1-50

GTTCTTAGAAGTTTAGAAGCTAGGGGTTGGTTTGATCAGCGAAAATAAAC

>24_MC06_537255_539352_-_12/1-50

GTTCTTAGGAGTTCTGGAGCTTTGGTTTGGCTTGATGAGCGAAAATAAAT

>12_W101_529263_531270_-_12/1-50

GTTCTTAGAAGTTTAGAGGTTTGGGGTTGGTCTGATCGGCGAAAATAAAC

>8_S6_457642_459625_+_12/1-50

GTTCTTAGGAGTTCTGGGGTTTGGGGCTGGTTTGATCTGCGAAAATTAAG

>14_W101_769847_771884_-_12/1-50

GTTCTTAGAAGTTTAGGAGTTAGCGACTGCTCTGATCGGCGAAAATAAAT

>34_NC96_463816_465964_+_12/1-50

GTTCTTAGAAGTTTAGGAGTTAGCGACTGCTCTGATCAGCGAAAATAAAT

>47_F_258414_260535_+_12/1-50

GTTCTTAGAAGTTTAGGAGTTAGCGACTACTCTGATCAGCGAAAATAAAT

>0_NC96_576349_578986_-_12/1-50

GTTCTTAGGAGTTCTGTGGTCTGGGGTTGGTTTGATCAGCGAAAATAAAC

>8_Rhigh_823704_825642_-_12/1-50

GTTCTTAGGAGTTCTGGGGTTTGGGGCTGGTTTGATCAGTGAAAATTAAG

>8_F_256125_258078_+_12/1-50

GTTCTTAGGAGTTCTGGGGTTTGGGGCTGGTTTGATCAGTGAAAATTAAG

>33_CA06_460842_463020_+_12/1-50

---CTTAGAAGTTTAGAGGTTTGGGGCTCGTCTGATCGACGAAAATAAAC

3. Alignment of 344 upstream flanks of non-12-GAA (GAA)n

>3_VA94_560912_562973_-_11/1-50

AAATGTAGATCTGGTTTA-GATATAAATATCTACAGAATCCTTTTATTTTC-

>17_W101_536401_538531_-_6/1-50

--ATTGAGTTGTTGTATTTAATATATAAAATAGCTAAAATCCTTTAAAAATC

>64_NC08_775261_777337_-_4/1-50

-TATTATTCACTGGTATTAAGTTGAAAAATAAG-TAAAAGCCTTTGTTTTTC

>23_Rlow_270092_270668_+_7/1-50

AAATT-ATTTCTGA-ATTAACTGTAAAAACGAA-TGAAAGCTTTTGTTTTC-

>13_NC96_571466_573551_-_6/1-50

ATATT-ATTTCTGA-ATCAACTGTAAAAGCGAA-CGGAACCCTTTGTTTTC-

>17_NC95_544696_546790_-_11/1-50

--ATTGAGTTGTTGTATTTAATATATAAAATAGCTAAAATCCTTTAAAAATC

>48_W101_260641_262669_+_10/1-50

--ATTGAGTTGTTGTATTTAATATATAAAATAGCTAAAATCCTTTAAAAATC

>10_Rhigh_502397_504434_+_7/1-50

--ATTGAGTTGTTGTATTTAATATATAAAATAGCTAAAATCCTTTAAAAATC

>27_Rlow_500056_502192_+_15/1-50

ATAATTTGTGATGGTTTTAACTATAAAAACTCA-CAAAACCTTTTGTTTTC-

>24_Rhigh_277083_279180_+_8/1-50

-TAACTGTATGTGGTTTCAACTATAAAAACTTA-TAAAATCCTTTGTTTTTC

>15_CA06_446599_448672_+_10/1-50

ACATT--TCTCTGA-ATTTGTTATAAAAATTAG-TGAAACCCTTGATTTTC-

>4_Rlow_258271_260311_+_10/1-50

AATTGTATATTTGGTTTT-AATATAAATATCAACAAAA-CCTTTTGTTTTCC

>62_VA94_797027_799074_-_9/1-50

--ATTGAGTTGTTGTATCTAATATATAAAATAGCTAAAATCCTTTAAAAATC

>14_NC08_547033_549127_-_6/1-50

--ATTGAGTTGTTGTATTTAATATATAAAATAGCTAAAATCCTTTAAAAATC

>45_MC06_765767_768017_-_4/1-50

TAATCTGGTTATGGTTTAAGATATAAAAACTTACAAAA-CCTTTTATTTTC-

>34_Rhigh_483110_485333_+_10/1-50

--ATTTAGTTGTTGTATTCACTATATAAAACGGCTAAAAGCCTTTAAACATC

>30_CA06_797230_799332_-_4/1-50

AAATT-ATTTCTGA-ATCAACTGTAAAAACGAA-TGAAACCCTTTGTTTTTC

>3_NC08_544680_546741_-_5/1-50

AAATGTAGATCTGGTTTA-GATATAAATATCTACAGAATCCTTTTATTTTC-

>0_Rlow_253105_255361_+_8/1-50

ACATT--TCTCTGA-ATTTGTTATAAAAATTAT-TAAAACTTTTTATTTTC-

>23_Rhigh_255818_256400_+_8/1-50

--ATTTAGTTGTTGTATTCACTATATAAAATGGCTAAAAATCTTTAAACATC

>22_S6_806718_808674_-_4/1-50

AAATT-ATTTCTGA-ATTAACTGTAAAAACGAA-TGAAAGCTTTTGTTTTC-

>49_NC96_456843_458895_+_8/1-50

AAATT-ATTTCTGA-ATTAACTGTAAAAACGAA-CTAAACCCTTTGTTTTTC

>8_NY01_783167_785114_-_10/1-50

AAATT-ATTTCTGA-ATTAACTGTAAAAATGAA-CGAAACCTTTTGTTTTCC

>12_NC08_532333_534364_-_8/1-50

GAATTTAATTGTGGATTA-ACTACAAAAACATATAAAAACCTTTTGTTTTC-

>6_NC08_271015_273034_+_9/1-50

--ATTGAGTTGTTGTATTTAATATATAAAATAGCTAAAATCCTTTAGAAATC

>27_NC96_263622_265692_+_13/1-50

AAATT-ATTTTTGA-ATTAACTGTAAAAATGAA-TGAAACCTTTTGTTTTCC

>14_Rhigh_594931_596980_-_7/1-50

ATAAGTTGTGATGGTTTTAACTATAAAAACTCA-CAAAACCTTTTGTTTTC-

>47_W101_750416_752723_-_6/1-50

GAATTTAATTGTGGATTA-ACTACAAAAACATATAAAAACCTTAAGTTTTC-

>9_S6_808983_811068_-_10/1-50

AATTGTAGATTTGGTTTT-AATATAAATATCGATAAAA-CCTTTTGTTTTCC

>13_NC96_268420_270511_+_8/1-50

AAATT-ATTTCTGA-ATTAACTATAAAAACTCA-CAAAATCTTTTGTTTTC-

>42_NC95_786317_788363_-_7/1-50

--ATTGAGTTGTTGTATCTAATATATAAAATAGCTAAAATCCTTTAAAAATC

>3_NC95_781574_783635_-_6/1-50

AAATGTAGATCTGGTTTA-GATATAAATATCTACAGAATCCTTTTATTTTC-

>45_NY01_778152_780420_-_4/1-50

TAATCTGGTTATGGTTTAAGATATAAAAACTTACAAAA-CCTTTTATTTTC-

>21_Rlow_604390_606655_-_13/1-50

--ATTTAGTTGTTGTATTCACTATATAAAATGGCTAAAAACCTTTAAACATC

>45_CA06_789960_792210_-_5/1-50

TAATCTGGTTATGGTTTAAGATATAAAAACTTACAAAA-CCTTTTATTTTC-

>54_Rlow_739532_740123_-_5/1-50

----------TTAAAACTAGACGTAAAACAAATGAAATGCTTGCTGCACTTC

>50_S6_462439_464548_+_22/1-50

AAATT-ATTTCTGA-ACTAACTTTAAAAACATA-CAAAA-CCTTTATTTAGC

>49_NY01_554651_556703_-_9/1-50

AAATT-ATTTCTGA-ATTAACTGTAAAAACGAA-CTAAACCCTTTGTTTTTC

>27_MC06_258651_260721_+_9/1-50

AAATT-ATTTTTGA-ATTAACTGTAAAAATGAA-TGAAACCTTTTGTTTTCC

>52_MC06_556715_558794_-_11/1-50

----TAAGTAGTTGTAAGTACGATAAAAACAAGGTAAAACCTTTTGTTTTCC

>5_CA06_444268_446263_+_8/1-50

--ATTTAGTTGTTGCATTCACTATATAAAATGGCTAAAAACCTTTAAACATC

>2_MC06_271068_273087_+_13/1-50

--ATTGAGTTGTTGTATTTAATATATAAAATAGCTAAAATCCTTTAGAAATC

>12_MC06_532352_534359_-_14/1-50

GAATTTAATTGTGGATTA-ACTACAAAAACATATAAAAACCTTTTGTTTTC-

>20_CA06_265397_267836_+_7/1-50

AATTGTATTTGTGATTGA-AGTATAAATATCAATAAAA-CCTTTTGTTTTC-

>4_MC06_261060_263139_+_10/1-50

--ATTGAGTTGTTGTATTTAATATATAAAATAGCTAAAATCCTTTAAAAATC

>50_NC96_454447_456541_+_8/1-50

--ATTGAGTTGTTGTATTTAATATATAAAATAGCTAAAATCCTTTAAAAATC

>3_NY01_793317_795378_-_5/1-50

AAATGTAGATCTGGTTTA-GATATAAATATCTACAGAATCCTTTTATTTTC-

>29_NC95_539880_541977_-_5/1-50

AATCGTAGTTGTGATTGA-AGTATAAATATCAACAAAA-CCCTTTGTTTCTG

>12_S6_567664_569698_-_10/1-50

--ATTGAGTTGTTGTATTTAATATATAAAATAGCTAAAATCCTTTAAAAATC

>6_Rlow_291870_293976_+_8/1-50

--ATTGAGTTGTTGTATTTAATATATAAAATAGCTAAAATCCTTTAGAAATC

>12_F_502115_504104_+_13/1-50

AAATT-ATTTCTGA-ATCAGCTGTAAAAAAGAA-CGGAACCCTTTGTTTTC-

>31_Rlow_607028_609215_-_10/1-50

ACATT--TCTCTGA-ATTTGTTATAAAAATTAG-TGAAACCTTTGATTTTC-

>45_NC96_799285_801535_-_4/1-50

TAATCTGGTTATGGTTTAAGATATAAAAACTTACAAAA-CCTTTTATTTTC-

>13_VA94_279156_281247_+_10/1-50

AAATT-ATTTCTGA-ATTAACTATAAAAACTCA-CAAAATCTTTTGTTTTC-

>3_S6_264317_266366_+_16/1-50

GATCGTAGTTGTGGTTGA-AGTATAAATATCAACAAAA-CCCTTTGTTTCTC

>9_Rlow_828737_830864_-_9/1-50

AAATT-ATTTCTGA-ATTAACTGTAAAAACGAA-TGAAAGCTTTTGTTTTC-

>26_W101_255829_257911_+_7/1-50

ACATT--TCTCTGA-ATTTGTTATAAAAATTAT-TAAAACTTTTTATTTTC-

>13_NC95_542275_544360_-_14/1-50

ATATT-ATTTCTGA-ATCAACTGTAAAAGCGAA-CGGAACCCTTTGTTTTC-

>12_NC95_537546_539553_-_10/1-50

GAATTTAATTGTGGATTA-ACTACAAAAACATATAAAAACCTTTTGTTTTC-

>24_NC08_763341_765438_-_19/1-50

ACATT--TCTCTGA-ATTTGCTATAAAAATTAG-TGAAACCTTTGGTTTGC-

>9_CA06_799642_801799_-_9/1-50

--ATTGAGTTGTTGTATTTAATATATAAAATAGCTAAAATCCTTTAAAAATC

>46_NY01_569002_571054_-_15/1-50

----TAAGTAGTTGTAAGTACGATAAAAACAAGGTAAAACCTTTTGTTTTCC

>29_CA06_563633_565730_-_8/1-50

AATCGTAGTTGTGATTGA-AGTATAAATATCAACAAAA-CCCTTTGTTTCTG

>42_NC08_549429_551475_-_7/1-50

--ATTGAGTTGTTGTATCTAATATATAAAATAGCTAAAATCCTTTAAAAATC

>44_MC06_528257_529718_-_7/1-50

AAATT-ATTTCTGA-ATCAACTGTAAAAACGAA-CGAAACCCCTTGTTTTTC

>59_S6_801732_804117_-_6/1-50

ACATT--TCTCTGA-ATTTGCTATAAAAATTAG-TGAAACCTTTTATTTTC-

>46_NC95_788650_790726_-_4/1-50

-TATTATTCACTGGTATTAAGTTGAAAAATAAG-TAAAAGCCTTTGTTTTTC

>14_CA06_807474_809568_-_9/1-50

--ATTGAGTTGTTGTATTTAATATATAAAATAGCTAAAATCCTTTAAAAATC

>2_Rhigh_495106_497206_+_7/1-50

ACATT--TCTCTGA-ATTTGTTATAAAAATTAG-TGAAACCTTTGATTTTC-

>35_Rlow_492855_494841_+_13/1-50

--ATTGAGTTGTTGTATTTAATATATAAAATAACTAAAATCCTTTAAAAATA

>42_NC96_819194_821240_-_8/1-50

--ATTGAGTTGTTGTATCTAATATATAAAATAGCTAAAATTCTTTAAAAATC

>32_Rlow_265429_267433_+_15/1-50

TAATCTAGTTATGGTTTAAGATATAAAAACTTATAAAA-CCTTTTATTTTC-

>26_NY01_261177_263259_+_11/1-50

ACATT--TCTCTGA-ATTTGTTATAAAAATTAT-TAAAACTTTTTATTTTC-

>9_VA94_786841_788968_-_10/1-50

--ATTGAGTTGTTGTATTTAATATATAAAATAGCTAAAATCCTTTAAAAATC

>45_NC08_760772_763028_-_4/1-50

TAATCTGGTTATGGTTTAAGATATAAAAACTTACAAAA-CCTTTTATTTTC-

>10_NC96_471163_473353_+_9/1-50

AAATT-ATTTCTGA-ATCAACTGTAAAAACGAA-CGAAACCCTTTGTTTTC-

>33_Rhigh_480559_482746_+_10/1-50

-TAAAGATGTGCGGTTTTAACTATAAAAACTAA-TAAAACTCTTTGTTTTTA

>1_Rlow_260639_262661_+_11/1-50

ATAACTTGTGATGGTTTTAACTGTAAAAACTCA-TAAAATCTTTTGTTTTC-

>21_Rhigh_604235_606500_-_17/1-50

--ATTTAGTTGTTGTATTCACTATATAAAATGGCTAAAAACCTTTAAACATC

>42_NC96_584055_586101_-_11/1-50

--ATTGAGTTGTTGTATCTAATATATAAAATAGCTAAAATCCTTTAAAAATC

>27_W101_258232_260302_+_8/1-50

AAATT-ATTTTTGA-ATTAACTGTAAAAATGAA-TGAAACCTTTTGTTTTCC

>9_S6_459965_462074_+_11/1-50

ACATT--TCTCTGA-ATTTGCTACAAAAATTAG-TGAAACCTTTTATTTTC-

>39_CA06_456108_458109_+_16/1-50

-TCTTTATTTTTTGGTTTAAAAATAAAAAAC-GTTAAAACCCTTATTTTCTA

>61_VA94_779726_781823_-_6/1-50

ACATT--TCTCTGA-ATTTGCTATAAAAATTAG-TGAAACCTTTGGTTTGC-

>10_CA06_465755_467945_+_10/1-50

AAATT-ATTTCTGA-ATCAACTGTAAAAACGAA-CGAAACCCTTTGTTTTC-

>44_NC95_533448_534909_-_8/1-50

AAATT-ATTTCTGA-ATCAACTGTAAAAACGAA-CGAAACCCCTTGTTTTTC

>9_Rhigh_828531_830658_-_9/1-50

AAATT-ATTTCTGA-ATTAACTGTAAAAACGAA-TGAAAGCTTTTGTTTTC-

>29_Rlow_597443_599537_-_10/1-50

GAATTTAATTGTGGATTA-ACTAAAAAAAAATATAAAAACCTTTTGTTTTC-

>39_Rhigh_821368_823313_-_9/1-50

ACATT--TCTCTGA-ATTTGCTATAAAAATTGG-TGAAACCTTTTATTTTC-

>15_S6_584223_586272_-_15/1-50

AAATT-ATTTCTGA-ATCAACTGTAAAAACGAA-TAAAAGATTTTGTTTTC-

>22_Rhigh_830943_833016_-_7/1-50

ACATT--TCTCTGA-ATTTGTTATAAAAATTAG-TGAAACCCTTGATTTTC-

>13_NY01_273592_275683_+_6/1-50

--ATTGAGTTGTTGTATTTAATATATAAAATAGCTAAAATCCTTTAAAAATC

>4_Rlow_272459_274499_+_7/1-50

AATTGTATATTTGGTTTT-AATATAAATATCAACAAAA-CCTTTTGTTTTCC

>22_Rlow_831149_833216_-_7/1-50

ACATT--TCTCTGA-ATTTGTTATAAAAATTAG-TGAAACCCTTGATTTTC-

>4_VA94_276755_278834_+_11/1-50

--ATTGAGTTGTTGTATTTAATATATAAAATAGCTAAAATCCTTTAAAAATC

>4_NC96_266025_268104_+_8/1-50

--ATTGAGTTGTTGTATTTAATATATAAAATAGCTAAAATCCTTTAAAAATC

>12_NC96_566716_568723_-_16/1-50

GAATTTAATTGTGGATTA-ACTACAAAAACATATAAAAACCTTTTGTTTTC-

>47_CA06_787227_789636_-_10/1-50

GAATTTAATTGTGGATTA-ACTACAAAAACATATAAAAACCTTAAGTTTTC-

>5_Rhigh_833358_835329_-_13/1-50

AATTGTAGATTTGGTTTT-AATATAAATATCGATAAAA-CCTTTTGTTTTCC

>4_S6_266678_268718_+_9/1-50

AATTGTAGATTTGGTTTT-AATATAAATATCAACAAAA-CCTTTTGTTTTCC

>1_S6_261893_263936_+_4/1-50

AAATC-ATTTCTGA-ATTAACTGTAAAAACGAA-TGAAAGCTTTTGTTTTC-

>47_VA94_774526_776857_-_4/1-50

GAATTTAATTGTGGATTA-ACTACAAAAACATATAAAAACCTTAAGTTTTC-

>8_Rhigh_485674_487609_+_5/1-50

AATCGTAGTTGTGGTTGA-AGTATAAATATCAACAAAA-CCCTTTGTTTCTC

>44_W101_525147_526608_-_14/1-50

AAATT-ATTTCTGA-ATCAACTGTAAAAACGAA-CGAAACCCCTTGTTTTTC

>37_Rhigh_611077_611374_-_6/1-50

-AGATAAGTAGTTGTAAGTACGATAAAAACAAGGTAAAACCTTTTGTTTTC-

>20_Rhigh_286653_289041_+_7/1-50

AAATT-ATTTCTGA-ATAAACTGTAAAAACGAA-TAAAAACTTTTGTTTTC-

>13_VA94_553045_555130_-_11/1-50

ATATT-ATTTCTGA-ATCAACTGTAAAAGCGAA-CGGAACCCTTTGTTTTC-

>29_Rhigh_597294_599388_-_8/1-50

GAATTTAATTGTGGATTA-ACTAAAAAAAAATATAAAAACCTTTTGTTTTC-

>22_Rhigh_281864_283925_+_9/1-50

ACATT--TCTCTGA-ATTTGTTATAAAAATTAG-TGAAACCCTTGATTTTC-

>33_NY01_545118_547296_-_9/1-50

AAATT-ATTTCTGA-ATTAACTGTAAAAACGAA-TGAAAGCTTTTGTTTCC-

>34_S6_579190_581509_-_13/1-50

AATTGTATTTGTGATTGA-AGAATAAATATCAATAAAA-CCCTTTGTTTTC-

>38_Rlow_820753_821224_-_11/1-50

-ACTATTTTTTTTGAATTAATGACAAAAAAC-GCTAAAACCTTTTATTTCTA

>46_VA94_568030_570106_-_9/1-50

----TAAGTAGTTGTAAGTACGATAAAAACAAGGTAAAACCTTTTGTTTTCC

>30_NC95_773706_775809_-_5/1-50

AAATT-ATTTCTGA-ATCAACTGTAAAAACGAA-TGAAACCCTTTGTTTTTC

>17_F_499668_501783_+_9/1-50

TAATCTAGTTATGGTTTAAGATATAAAAACTTACAAAA-CCTTTTATTTTC-

>3_MC06_549600_551661_-_11/1-50

AAATGTAGATCTGGTTTA-GATATAAATATCTACAGAATCCTTTTATTTTC-

>16_Rlow_490471_492517_+_9/1-50

AAATT-ATTTCTGA-ATCAACTGTAAAAACGAA-TAAAAGATTTTGTTTTC-

>24_F_497265_499338_+_13/1-50

AATCGTAGTTGTGGTCGA-AGTATAAATATCAACAAAA-CCCTTTTTTTATC

>42_NY01_798057_800103_-_7/1-50

--ATTGAGTTGTTGTATCTAATATATAAAATAGCTAAAATCCTTTAAAAATC

>6_W101_267695_269714_+_8/1-50

--ATTGAGTTGTTGTATTTAATATATAAAATAGCTAAAATCCTTTAGAAATC

>3_CA06_805118_807179_-_6/1-50

AAATGTAGATCTGGTTTA-GATATAAATATCTACAGAATCCTTTTATTTTC-

>20_NC96_270809_273248_+_6/1-50

AATTGTATTTGTGATTGA-AGTATAAATATCAATAAAA-CCTTTTGTTTTC-

>36_Rhigh_497529_499458_+_11/1-50

-TAACTGTATGTGGTTTCAACTATAAAAACTTA-TAAAATCCTTTGTTTTTC

>13_NY01_268402_270493_+_16/1-50

AAATT-ATTTCTGA-ATTAACTATAAAAACTCA-CAAAATCTTTTGTTTTC-

>17_NC08_539528_541628_-_16/1-50

--ATTGAGTTGTTGTATTTAATATATAAAATAGCTAAAATCCTTTAAAAATC

>51_NC96_447141_449379_+_9/1-50

-TAAAGATGTGCGGTTTTAACTATAAAAACTAA-TAAAACTCTTTGTTTTTA

>0_F_248320_250951_+_10/1-50

ACATT--TCTCTGA-ATTTGTTATAAAAATTAT-TAAAACTTTTTATTTTC-

>46_VA94_799367_801443_-_4/1-50

-TATTATTCACTGGTATTAAGTTGAAAAATAAG-TAAAAGCCTTTGTTTTTC

>20_Rlow_286739_289127_+_7/1-50

AAATT-ATTTCTGA-ATAAACTGTAAAAACGAA-TAAAAACTTTTGTTTTC-

>39_NC96_461504_463505_+_11/1-50

-TCTTTATTTTTTGGTTTAAAAATAAAAAAC-GTTAAAACCCTTATTTTCTA

>17_CA06_568458_570564_-_7/1-50

--ATTGAGTTGTTGTATTTAATATATAAAATAGCTAAAATCCTTTAAAAATC

>16_NC96_273615_275724_+_6/1-50

--ATTGAGTTGTTGTATTTAATATATAAAATAGCTAAAATCCTTTAAAAATC

>15_S6_813835_815878_-_16/1-50

TAATCTAGTTATGGTTTAAGATATAAAAACTTACAAAA-GCTTTTATTTTC-

>22_Rlow_267768_269823_+_10/1-50

ACATT--TCTCTGA-ATTTGTTATAAAAATTAG-TGAAACCCTTGATTTTC-

>13_CA06_263005_265096_+_6/1-50

AAATT-ATTTCTGA-ATTAACTATAAAAACTCA-CAAAATCTTTTGTTTTC-

>4_F_795960_797976_-_14/1-50

AATTGTAGATTTGGTTTT-AATATAAATATCAACAAAA-CCTTTTGTTTTCC

>45_VA94_777163_779413_-_4/1-50

TAATCTGGTTATGGTTTAAGATATAAAAACTTACAAAA-CCTTTTATTTTC-

>6_CA06_270567_272586_+_9/1-50

--ATTGAGTTGTTGTATTTAATATATAAAATAGCTAAAATCCTTTAGAAATC

>29_NC96_569068_571165_-_6/1-50

AATCGTAGTTGTGATTGA-AGTATAAATATCAACAAAA-CCCTTTGTTTCTG

>23_S6_812654_813461_-_19/1-50

AAATT--TTTATGA-ATTAACATAAAAAATCGT-CAAAATCCTTTATTTCTA

>33_Rlow_480651_482838_+_9/1-50

-TAAAGATGTGCGGTTTTAACTATAAAAACTAA-TAAAACTCTTTGTTTTTA

>32_Rhigh_279528_281532_+_15/1-50

TAATCTAGTTATGGTTTAAGATATAAAAACTTATAAAA-CCTTTTATTTTC-

>25_Rlow_838234_840358_-_7/1-50

-------CATTTTACATTTAGTCAAAAAACCGA-CGAAAGTCTTTGTTTATA

>6_VA94_286763_288782_+_11/1-50

--ATTGAGTTGTTGTATTTAATATATAAAATAGCTAAAATCCTTTAGAAATC

>13_NC95_268424_270515_+_6/1-50

AAATT-ATTTCTGA-ATTAACTATAAAAACTCA-CAAAATCTTTTGTTTTC-

>13_NC08_537107_539192_-_14/1-50

ATATT-ATTTCTGA-ATCAACTGTAAAAGCGAA-CGGAACCCTTTGTTTTC-

>12_NY01_542729_544739_-_15/1-50

--ATTTAGTTGTTGTATTCACTATATAAAATGGCTAAAAACCTTTAAACATC

>13_CA06_566037_568122_-_14/1-50

ATATT-ATTTCTGA-ATCAACTGTAAAAGCGAA-CGGAACCCTTTGTTTTC-

>39_Rlow_821569_823510_-_8/1-50

ACATT--TCTCTGA-ATTTGCTATAAAAATTGG-TGAAACCTTTTATTTTC-

>4_W101_543944_545993_-_10/1-50

----TAAGTAGTTGTAAGTACGATAAAAACAAGGTAAAACCTTTTGTTTTCC

>52_W101_767449_769519_-_10/1-50

AAATT-ATTTCTGA-ATCAACTGTAAAAACGAA-TGAAACCCTTTGTTTTTC

>13_S6_581827_583894_-_10/1-50

--ATTGAGTTGTTGTATTTAATATATAAAATAACTAAAATCCTTTAAAAATA

>23_Rlow_255877_256471_+_10/1-50

--ATTTAGTTGTTGTATTCACTATATAAAATGGCTAAAAATCTTTAAACATC

>15_Rhigh_289417_291490_+_8/1-50

-----GAGTTGTTGTATTTAATATATAAAATAGCTAAAATCCTTTAAAAATC

>40_Rhigh_826006_828211_-_9/1-50

AAATT-ATTTCTGA-ATTAATTGCAAAAATGAA-TGAAACCTTTTGTTTGC-

>47_NC08_758243_760466_-_4/1-50

GAATTTAATTGTGGATTA-ACTACAAAAACATATAAAAACCTTAAGTTTTC-

>5_NY01_561871_563866_-_9/1-50

--ATTTAGTTGTTGCATTCACTATATAAAATGGCTAAAAACCTTTAAACATC

>44_S6_562996_565003_-_16/1-50

AAATT-ATTTCTGA-ATCAACTGTAAAAACGAA-CGAAACCCCTTGTTTTTC

>26_CA06_255804_257886_+_4/1-50

ACATT--TCTCTGA-ATTTGTTATAAAAATTAT-TAAAACTTTTTATTTTC-

>17_Rlow_488036_490148_+_11/1-50

ACATT--TCTCTGA-ATTTGTTATAAAAATTAG-TGAAACCTTTGATTTTC-

>50_NY01_557008_559102_-_18/1-50

--ATTGAGTTGTTGTATTTAATATATAAAATAGCTAAAATCCTTTAAAAATC

>9_NC96_808984_811111_-_11/1-50

--ATTGAGTTGTTGTATTTAATATATAAAATAGCTAAAATCCTTTAAAAATC

>47_NC96_796558_798979_-_4/1-50

GAATTTAATTGTGGATTA-ACTACAAAAACATATAAAAACCTTAAGTTTTC-

>14_NC95_552480_554574_-_9/1-50

--ATTGAGTTGTTGTATTTAATATATAAAATAGCTAAAATCCTTTAAAAATC

>42_NC95_554885_556931_-_7/1-50

--ATTGAGTTGTTGTATCTAATATATAAAATAGCTAAAATCCTTTAAAAATC

>9_MC06_775500_777585_-_5/1-50

--ATTGAGTTGTTGTATTTAATATATAAAATAGCTAAAATCCTTTAAAAATC

>36_CA06_463384_465280_+_10/1-50

--ATTTAGTTGTTGTATTCACTATATAAAATGGCTAAAAACCTTTAAACATC

>17_S6_586613_588743_-_11/1-50

--ATTGAGTTGTTGTATTTAATATATAAAATAGCTAAAATCCTTTAAAAATC

>44_NY01_538634_540095_-_7/1-50

AAATT-ATTTCTGA-ATCAACTGTAAAAACGAA-CGAAACCCCTTGTTTTTC

>26_F_253707_255777_+_8/1-50

--ATTGAGTTGTTGTATTTAATATATAAAACAGCTAAAATCCTTTAGAAATC

>1_Rlow_274827_276849_+_11/1-50

ATAACTTGTGATGGTTTTAACTGTAAAAACTCA-TAAAATCTTTTGTTTTC-

>23_MC06_268674_270759_+_8/1-50

--ATTGAGTTGTTGTATTTAATATATAAAATAGCTAAAATCCTTTAAAAATC

>24_NY01_780733_782830_-_11/1-50

ACATT--TCTCTGA-ATTTGCTATAAAAATTAG-TGAAACCTTTGGTTTGC-

>6_Rhigh_291778_293884_+_6/1-50

--ATTGAGTTGTTGTATTTAATATATAAAATAGCTAAAATCCTTTAGAAATC

>15_NC95_273625_275710_+_6/1-50

--ATTGAGTTGTTGTATTTAATATATAAAATAGCTAAAATCCTTTAAAAATC

>12_VA94_548313_550320_-_9/1-50

GAATTTAATTGTGGATTA-ACTACAAAAACATATAAAAACCTTTTGTTTTC-

>22_Rlow_281938_284005_+_9/1-50

ACATT--TCTCTGA-ATTTGTTATAAAAATTAG-TGAAACCCTTGATTTTC-

>20_VA94_281557_283996_+_10/1-50

AATTGTATTTGTGATTGA-AGTATAAATATCAATAAAA-CCTTTTGTTTTC-

>30_NY01_785456_787559_-_5/1-50

AAATT-ATTTCTGA-ATCAACTGTAAAAACGAA-TGAAACCCTTTGTTTTTC

>26_NC95_261229_263311_+_6/1-50

ACATT--TCTCTGA-ATTTGTTATAAAAATTAT-TAAAACTTTTTATTTTC-

>6_S6_271453_273514_+_22/1-50

AATTT--TCTCTGA-ATTTACTATGAAAATTAG-TGAAACCCTTGATTTTC-

>1_Rhigh_274747_276769_+_9/1-50

ATAACTTGTGATGGTTTTAACTGTAAAAACTCA-TAAAATCTTTTGTTTTC-

>0_CA06_802173_804810_-_8/1-50

AATTGTAGATTTGGTTTT-AATATAAATATCAACAAAA-CCTTTTGTTTTCC

>20_MC06_265862_268301_+_16/1-50

AATTGTATTTGTGATTGA-AGTATAAATATCAATAAAA-CCTTTTGTTTTC-

>26_VA94_271916_273998_+_10/1-50

ACATT--TCTCTGA-ATTTGTTATAAAAATTAT-TAAAACTTTTTATTTTC-

>3_NC96_579306_581367_-_8/1-50

AAATGTAGATCTGGTTTA-GATATAAATATCTACAGAATCCTTTTATTTTC-

>47_S6_798710_800825_-_11/1-50

-ACTATTTTTTTTGAATTAATGACAAAAAAC-GCTAAAACCTTTTATTTCTA

>42_MC06_785368_787414_-_9/1-50

-----------TTGTATCTAATATATAAAATAGCTAAAATCCTTTAAAAATC

>42_VA94_565700_567746_-_6/1-50

--ATTGAGTTGTTGTATCTAATATATAAAATAGCTAAAATCCTTTAAAAATC

>42_CA06_578362_580408_-_13/1-50

--ATTGAGTTGTTGTATCTAATATATAAAATAGCTAAAATCCTTTAAAAATC

>4_CA06_260616_262695_+_9/1-50

--ATTGAGTTGTTGTATTTAATATATAAAATAGCTAAAATCCTTTAAAAATC

>24_NC95_768977_771074_-_13/1-50

ACATT--TCTCTGA-ATTTGCTATAAAAATTAG-TGAAACCTTTGGTTTGC-

>8_S6_804441_806394_-_14/1-50

AAATTGATTTCTGA-ACTAACTTTAAAAACATA-CAAAA-CCTTTATTTAGC

>0_Rhigh_253052_255308_+_8/1-50

ACATT--TCTCTGA-ATTTGTTATAAAAATTAT-TAAAACTTTTTATTTTC-

>47_NC95_763720_766105_-_4/1-50

GAATTTAATTGTGGATTA-ACTACAAAAACATATAAAAACCTTAAGTTTTC-

>24_Rhigh_262901_264998_+_8/1-50

-TAACTGTATGTGGTTTCAACTATAAAAACTTA-TAAAATCCTTTGTTTTTC

>24_CA06_792526_794623_-_8/1-50

ACATT--TCTCTGA-ATTTGCTATAAAAATTAG-TGAAACCTTTGGTTTGC-

>17_VA94_555457_557575_-_11/1-50

--ATTGAGTTGTTGTATTTAATATATAAAATAGCTAAAATCCTTTAAAAATC

>3_MC06_780598_782659_-_9/1-50

AAATGTAGATCTGGTTTA-GATATAAATATCTACAGAATCCTTTTATTTTC-

>3_NC96_814427_816488_-_6/1-50

AAATGTAGATCTGGTTTA-GATATAAATATCTACAGAATCCTTTTATTTTC-

>42_W101_772204_774250_-_8/1-50

--ATTGAGTTGTTGTATCTAATATATAAAATAGCTAAAATCCTTTAAAAATC

>4_NC08_261052_263131_+_8/1-50

--ATTGAGTTGTTGTATTTAATATATAAAATAGCTAAAATCCTTTAAAAATC

>55_NY01_559440_561525_-_13/1-50

ACATT--TCTCTGA-ATTTGTTATAAAAATTAG-TGAAACCCTTGATTTTC-

>0_MC06_546943_549289_-_9/1-50

AATTGTAGATTTGGTTTT-AATATAAATATCAACAAAA-CCTTTTGTTTTCC

>46_NC96_821530_823606_-_4/1-50

-TATTATTCACTGGTATTAAGTTGAAAAATAAG-TAAAAGCCTTTGTTTTTC

>30_VA94_784431_786516_-_9/1-50

AAATT-ATTTCTGA-ATCAACTGTAAAAACGAA-TGAAACCCTTTGTTTTTC

>12_Rhigh_592634_594635_-_10/1-50

AAATGTAGATCTGGTTTA-GATATAAATATTTACAGAATTCTTTTATTTTC-

>48_NY01_552318_554346_-_9/1-50

ATAAGTTGTGATGGTTTTAACTATAAAAACTGA-TAAAATCTTTTGTTTTC-

>8_NC08_765799_767746_-_8/1-50

AAATT-ATTTCTGA-ATTAACTGTAAAAATGAA-CGAAACCTTTTGTTTTCC

>5_NC96_449732_451727_+_6/1-50

--ATTTAGTTGTTGCATTCACTATATAAAATGGCTAAAAACCTTTAAACATC

>34_CA06_458414_460544_+_10/1-50

--ATTGAGTTGTTGTATTTAATATATAAAATAGCTAAAATCCTTTAAAAATC

>13_W101_533995_536080_-_9/1-50

ATATT-ATTTCTGA-ATCAACTGTAAAAGCGAA-CGGAACCCTTTGTTTTC-

>24_Rlow_262984_265081_+_11/1-50

-TAACTGTATGTGGTTTCAACTATAAAAACTTA-TAAAATCCTTTGTTTTTC

>22_F_251355_253422_+_19/1-50

--ATTGAGTTGTTGTATTTAATATATAAAATAGCTAAAATCCTTTAAAAATC

>44_NC96_562609_564070_-_11/1-50

AAATT-ATTTCTGA-ATCAACTGTAAAAACGAA-CGAAACCCCTTGTTTTTC

>4_Rhigh_272385_274425_+_8/1-50

AATTGTATATTTGGTTTT-AATATAAATATCAACAAAA-CCTTTTGTTTTCC

>24_Rlow_277172_279269_+_11/1-50

-TAACTGTATGTGGTTTCAACTATAAAAACTTA-TAAAATCCTTTGTTTTTC

>44_CA06_557210_558671_-_7/1-50

AAATT-ATTTCTGA-ATCAACTGTAAAAACGAA-CGAAACCCCTTGTTTTTC

>3_W101_541559_543620_-_10/1-50

AAATT-ATTTCTGA-ATTAACTATAAAAACTCA-CAAAATCTTTTGTTTTC-

>20_NY01_270788_273227_+_5/1-50

AATTGTATTTGTGATTGA-AGTATAAATATCAATAAAA-CCTTTTGTTTTC-

>34_Rlow_483196_485419_+_8/1-50

--ATTTAGTTGTTGTATTCACTATATAAAACGGCTAAAAGCCTTTAAACATC

>0_MC06_777947_780290_-_8/1-50

AATTGTAGATTTGGTTTT-AATATAAATATCAACAAAA-CCTTTTGTTTTCC

>47_NY01_775551_777846_-_4/1-50

GAATTTAATTGTGGATTA-ACTACAAAAACATATAAAAACCTTAAGTTTTC-

>6_NC95_276007_278026_+_9/1-50

--ATTGAGTTGTTGTATTTAATATATAAAATAGCTAAAATCCTTTAGAAATC

>10_Rlow_502531_504568_+_7/1-50

--ATTGAGTTGTTGTATTTAATATATAAAATAGCTAAAATCCTTTAAAAATC

>26_NC08_256246_258328_+_8/1-50

ACATT--TCTCTGA-ATTTGTTATAAAAATTAT-TAAAACTTTTTATTTTC-

>3_VA94_792281_794342_-_7/1-50

AAATGTAGATCTGGTTTA-GATATAAATATCTACAGAATCCTTTTATTTTC-

>30_W101_760339_762443_-_10/1-50

AAATT-ATTTCTGA-ATCAACTGTAAAAACGAA-TGAAACCCTTTGTTTTTC

>20_NY01_275988_278427_+_9/1-50

AATTGTATTTGTGATTGA-AGTATAAATATCAATAAAA-CCTTTTGTTTTC-

>46_S6_259528_261616_+_9/1-50

--ATTGAGTTGTTGTATTTAATATATAAAATAGCTAAAATCCTTTAAAAATC

>29_VA94_550644_552741_-_7/1-50

AATCGTAGTTGTGATTGA-AGTATAAATATCAACAAAA-CCCTTTGTTTCTG

>3_CA06_573598_575659_-_6/1-50

AAATGTAGATCTGGTTTA-GATATAAATATCTACAGAATCCTTTTATTTTC-

>38_CA06_809879_811925_-_13/1-50

--ATTGAGTTGTTGTATCTAATATATAAAATAGCTAAAATCCTTTAAAAATC

>15_Rlow_289503_291576_+_8/1-50

-----GAGTTGTTGTATTTAATATATAAAATAGCTAAAATCCTTTAAAAATC

>5_S6_269082_271062_+_23/1-50

ATAACTTGTGATGGTTTTAACTGTAAAAACTCA-TAAAATCTTTTGTTTTC-

>29_NC08_534685_536782_-_14/1-50

AATCGTAGTTGTGATTGA-AGTATAAATATCAACAAAA-CCCTTTGTTTCTG

>27_NC95_263632_265702_+_8/1-50

AAATT-ATTTTTGA-ATTAACTGTAAAAATGAA-TGAAACCTTTTGTTTTCC

>0_VA94_789345_791982_-_5/1-50

AATTGTAGATTTGGTTTT-AATATAAATATCAACAAAA-CCTTTTGTTTTCC

>56_NY01_564228_566340_-_14/1-50

--ATTGAGTTGTTGTATTTAATATATAAAATAGCTAAAATCCTTTAAAAATC

>37_Rlow_611232_611529_-_7/1-50

-AGATAAGTAGTTGTAAGTACGATAAAAACAAGGTAAAACCTTTTGTTTTC-

>48_CA06_453786_455814_+_17/1-50

ATAAGTTGTGATGGTTTTAACTATAAAAACTGA-TAAAATCTTTTGTTTTC-

>40_Rlow_826200_828405_-_13/1-50

AAATT-ATTTCTGA-ATTAATTGCAAAAATGAA-TGAAACCTTTTGTTTGC-

>45_MC06_534698_536942_-_4/1-50

TAATCTGGTTATGGTTTAAGATATAAAAACTTACAAAA-CCTTTTATTTTC-

>46_NC96_586400_588476_-_10/1-50

----TAAGTAGTTGTAAGTACGATAAAAACAAGGTAAAACCTTTTGTTTTCC

>26_MC06_256245_258327_+_9/1-50

ACATT--TCTCTGA-ATTTGTTATAAAAATTAT-TAAAACTTTTTATTTTC-

>15_NC96_452067_454140_+_11/1-50

ACATT--TCTCTGA-ATTTGTTATAAAAATTAG-TGAAACCCTTGATTTTC-

>5_Rlow_833558_835529_-_9/1-50

AATTGTAGATTTGGTTTT-AATATAAATATCGATAAAA-CCTTTTGTTTTCC

>32_Rlow_279602_281606_+_10/1-50

TAATCTAGTTATGGTTTAAGATATAAAAACTTATAAAA-CCTTTTATTTTC-

>36_NC96_468795_470691_+_7/1-50

--ATTTAGTTGTTGTATTCACTATATAAAATGGCTAAAAACCTTTAAACATC

>3_F_798303_800352_-_9/1-50

GATCGTAGTTGTGGTTGA-AGTATAAATATCAACAAAA-CCCTTTGTTTCTC

>46_F_805439_807527_-_4/1-50

-TATTATTCACTGGTATTAAGTTGAAAAATAAG-TAAAAGCCTTTGTTTTTC

>14_CA06_575954_578048_-_10/1-50

--ATTGAGTTGTTGTATTTAATATATAAAATAGCTAAAATCCTTTAAAAATC

>14_W101_762771_764808_-_8/1-50

--ATTGAGTTGTTGTATTTAATATATAAAATAGCTAAAATCCTTTAAAAATC

>30_S6_455211_457326_+_17/1-50

-TAAAGATGTGCGGTTTTAACTATAAAAACTAA-TAAAACTCTTTGTTTTTA

>44_VA94_544218_545679_-_7/1-50

AAATT-ATTTCTGA-ATCAACTGTAAAAACGAA-CGAAACCCCTTGTTTTTC

>8_CA06_794951_796898_-_10/1-50

AAATT-ATTTCTGA-ATTAACTGTAAAAATGAA-CGAAACCTTTTGTTTTCC

>43_NY01_550041_552042_-_10/1-50

-TCTTTATTTTTTGGTTTAAAAATAAAAAAC-GTTAAAACCCTTATTTTCTA

>26_Rlow_284290_286423_+_9/1-50

AAATT-ATTTCTGA-ATTAACTGTAAAAACGAA-TGAAAGCTTTTGTTTTC-

>34_NY01_547585_549733_-_11/1-50

--ATTGAGTTGTTGTATTTAATATATAAAATAGCTAAAATCCTTTAAAAATC

>13_MC06_263443_265534_+_4/1-50

AAATT-ATTTCTGA-ATTAACTATAAAAACTCA-CAAAATCTTTTGTTTTC-

>3_NC95_550115_552176_-_9/1-50

AAATGTAGATCTGGTTTA-GATATAAATATCTACAGAATCCTTTTATTTTC-

>49_CA06_451405_453457_+_8/1-50

AAATT-ATTTCTGA-ATTAACTGTAAAAACGAA-CTAAACCCTTTGTTTTTC

>8_NC95_771417_773364_-_10/1-50

AAATT-ATTTCTGA-ATTAACTGTAAAAATGAA-CGAAACCTTTTGTTTTCC

>8_Rlow_485760_487695_+_5/1-50

AATCGTAGTTGTGGTTGA-AGTATAAATATCAACAAAA-CCCTTTGTTTCTC

>9_NC95_776122_778249_-_11/1-50

--ATTGAGTTGTTGTATTTAATATATAAAATAGCTAAAATCCTTTAAAAATC

>20_NC08_265839_268278_+_8/1-50

AATTGTATTTGTGATTGA-AGTATAAATATCAATAAAA-CCTTTTGTTTTC-

>12_Rlow_592768_594769_-_11/1-50

AAATGTAGATCTGGTTTA-GATATAAATATTTACAGAATTCTTTTATTTTC-

>44_NC08_528226_529687_-_11/1-50

AAATT-ATTTCTGA-ATCAACTGTAAAAACGAA-CGAAACCCCTTGTTTTTC

>26_NC96_261204_263286_+_9/1-50

ACATT--TCTCTGA-ATTTGTTATAAAAATTAT-TAAAACTTTTTATTTTC-

>27_Rhigh_499922_502058_+_9/1-50

ATAATTTGTGATGGTTTTAACTATAAAAACTCA-CAAAACCTTTTGTTTTC-

>47_MC06_763220_765461_-_4/1-50

GAATTTAATTGTGGATTA-ACTACAAAAACATATAAAAACCTTAAGTTTTC-

>45_W101_753035_755309_-_5/1-50

TAATCTGGTTATGGTTTAAGATATAAAAACTTACAAAA-CCTTTTATTTTC-

>1_F_800711_802754_-_9/1-50

GATCGTAGTTGTGGTTGA-AGTATAAATATCAACAAAA-CCCTTTGTTTCTC

>34_W101_265262_267401_+_8/1-50

--------TTGTTGTATTTAATATATAAAATAGCTAAAATCCTTTAAAAATC

>6_F_791276_793289_-_13/1-50

AAATT--TCTCTGA-ATTTACTATGAAAATTAG-TGAAACCCTTGATTTTC-

>42_S6_589038_591120_-_18/1-50

----TAAGTAGTTGTAAGTACGATAAAAACAAGGTAAAACCTTTTGTTTTCC

>58_S6_569986_572158_-_18/1-50

AAATT-ATTTCTGA-ATCAACTGTAAAAACGAA-CGAAACCCCTTGTTTTTC

>0_NC08_542023_544366_-_10/1-50

AATTGTAGATTTGGTTTT-AATATAAATATCAACAAAA-CCTTTTGTTTTCC

>10_S6_464867_466910_+_14/1-50

AAATT-ATTTCTGA-ATTAACTGTAAAAACGAA-CTAAACCCTTTGTTTTC-

>4_NY01_265995_268062_+_8/1-50

--ATTGAGTTGTTGTATTTAATATATAAAATAGCTAAAATCCTTTAAAAATC

>4_Rhigh_258200_260240_+_10/1-50

AATTGTATATTTGGTTTT-AATATAAATATCAACAAAA-CCTTTTGTTTTCC

>30_MC06_773065_775168_-_8/1-50

AAATT-ATTTCTGA-ATCAACTGTAAAAACGAA-TGAAACCCTTTGTTTTTC

>16_Rhigh_490409_492455_+_20/1-50

AAATT-ATTTCTGA-ATCAACTGTAAAAACGAA-TAAAAGATTTTGTTTTC-

>26_Rhigh_284210_286337_+_9/1-50

AAATT-ATTTCTGA-ATTAACTGTAAAAACGAA-TGAAAGCTTTTGTTTTC-

>42_MC06_554358_556404_-_15/1-50

--ATTGAGTTGTTGTATCTAATATATAAAATAGCTAAAATCCTTTAAAAATC

>43_W101_263010_264957_+_11/1-50

-TCTTTATTTTTTGGTTTAAAAATAAAAAAC-GTTAAAACCCTTATTTTCTA

>35_Rhigh_492796_494782_+_14/1-50

--ATTGAGTTGTTGTATTTAATATATAAAATAACTAAAATCCTTTAAAAATA

>46_NC95_557218_559294_-_11/1-50

----TAAGTAGTTGTAAGTACGATAAAAACAAGGTAAAACCTTTTGTTTTCC

>17_NC96_573863_575969_-_11/1-50

--ATTGAGTTGTTGTATTTAATATATAAAATAGCTAAAATCCTTTAAAAATC

>8_MC06_539692_541639_-_14/1-50

AAATT-ATTTCTGA-ATTAACTGTAAAAATGAA-CGAAACCTTTTGTTTTCC

>17_Rhigh_487941_490053_+_8/1-50

ACATT--TCTCTGA-ATTTGTTATAAAAATTAG-TGAAACCTTTGATTTTC-

>31_Rhigh_606885_609072_-_6/1-50

ACATT--TCTCTGA-ATTTGTTATAAAAATTAG-TGAAACCTTTGATTTTC-

>0_NC95_778629_781266_-_8/1-50

AATTGTAGATTTGGTTTT-AATATAAATATCAACAAAA-CCTTTTGTTTTCC

>24_MC06_768330_770427_-_10/1-50

ACATT--TCTCTGA-ATTTGCTATAAAAATTAG-TGAAACCTTTGGTTTGC-

>8_NC96_804273_806220_-_8/1-50

AAATT-ATTTCTGA-ATTAACTGTAAAAATGAA-CGAAACCTTTTGTTTTCC

>0_NC95_547170_549807_-_8/1-50

AATTGTAGATTTGGTTTT-AATATAAATATCAACAAAA-CCTTTTGTTTTCC

>8_VA94_782145_784092_-_9/1-50

AAATT-ATTTCTGA-ATTAACTGTAAAAATGAA-CGAAACCTTTTGTTTTCC

>14_NC96_816783_818877_-_11/1-50

--ATTGAGTTGTTGTATTTAATATATAAAATAGCTAAAATCCTTTAAAAATC

>12_CA06_561305_563312_-_8/1-50

GAATTTAATTGTGGATTA-ACTACAAAAACATATAAAAACCTTTTGTTTTC-

>15_NY01_278800_280873_+_8/1-50

--ATTGAGTTGTTGTATTTAATATATAAAATAGCTAAAATCCTTTAAAAATC

>2_Rlow_495168_497274_+_8/1-50

ACATT--TCTCTGA-ATTTGTTATAAAAATTAG-TGAAACCTTTGATTTTC-

>0_S6_256524_259155_+_8/1-50

ACATT--TCTCTGA-ATTTGTTATAAAAATTAT-TAAAACTTTTTATTTTC-

>50_CA06_449009_451103_+_18/1-50

--ATTGAGTTGTTGTATTTAATATATAAAATAGCTAAAATCCTTTAAAAATC

>6_NC96_276045_278064_+_17/1-50

--ATTGAGTTGTTGTATTTAATATATAAAATAGCTAAAATCCTTTAGAAATC

>46_CA06_812230_814282_-_4/1-50

-TATTATTCACTGGTATTAAGTTGAAAAATAAG-TAAAAGCCTTTGTTTTTC

>30_NC96_806556_808659_-_9/1-50

AAATT-ATTTCTGA-ATCAACTGTAAAAACGAA-TGAAACCCTTTGTTTTTC

>42_W101_765116_767162_-_7/1-50

--ATTGAGTTGTTGTATCTAATATATAAAATAGCTAAAATCCTTTAAAAATC

>27_VA94_274343_276413_+_16/1-50

AAATT-ATTTTTGA-ATTAACTGTAAAAATGAA-TGAAACCTTTTGTTTTCC

>24_W101_755625_757722_-_8/1-50

ACATT--TCTCTGA-ATTTGCTATAAAAATTAG-TGAAACCTTTGGTTTGC-

>23_Rhigh_270003_270591_+_6/1-50

AAATT-ATTTCTGA-ATTAACTGTAAAAACGAA-TGAAAGCTTTTGTTTTC-

>60_S6_816229_818317_-_7/1-50

-------CATTTTACATTTAGTCAAAAAACCGA-CGAAAGTCTTTGTTTATA

>0_NC96_811491_814128_-_5/1-50

AATTGTAGATTTGGTTTT-AATATAAATATCAACAAAA-CCTTTTGTTTTCC

>13_NC08_263444_265535_+_7/1-50

AAATT-ATTTCTGA-ATTAACTATAAAAACTCA-CAAAATCTTTTGTTTTC-

>0_CA06_570932_573275_-_13/1-50

AATTGTAGATTTGGTTTT-AATATAAATATCAACAAAA-CCTTTTGTTTTCC

>42_NY01_566666_568712_-_8/1-50

--ATTGAGTTGTTGTATCTAATATATAAAATAGCTAAAATCCTTTAAAAATC

>14_VA94_563283_565377_-_13/1-50

--ATTGAGTTGTTGTATTTAATATATAAAATAGCTAAAATCCTTTAAAAATC

>46_CA06_580713_582789_-_6/1-50

----TAAGTAGTTGTAAGTACGATAAAAACAAGGTAAAACCTTTTGTTTTCC

>24_NC96_801848_803945_-_8/1-50

ACATT--TCTCTGA-ATTTGCTATAAAAATTAG-TGAAACCTTTGGTTTGC-

>1_Rhigh_260565_262587_+_10/1-50

ATAACTTGTGATGGTTTTAACTGTAAAAACTCA-TAAAATCTTTTGTTTTC-

>8_W101_758050_759997_-_10/1-50

AAATT-ATTTCTGA-ATTAACTGTAAAAATGAA-CGAAACCTTTTGTTTTCC

>27_CA06_258210_260280_+_9/1-50

AAATT-ATTTTTGA-ATTAACTGTAAAAATGAA-TGAAACCTTTTGTTTTCC

>14_MC06_782963_785057_-_9/1-50

--ATTGAGTTGTTGTATTTAATATATAAAATAGCTAAAATCCTTTAAAAATC

>36_Rlow_497645_499574_+_27/1-50

-TAACTGTATGTGGTTTCAACTATAAAAACTTA-TAAAATCCTTTGTTTTTC

>27_NC08_258649_260719_+_8/1-50

AAATT-ATTTTTGA-ATTAACTGTAAAAATGAA-TGAAACCTTTTGTTTTCT

>20_NC95_270819_273258_+_8/1-50

AATTGTATTTGTGATTGA-AGTATAAATATCAATAAAA-CCTTTTGTTTTC-

>48_NC96_459197_461225_+_8/1-50

ATAAGTTGTGATGGTTTTAACTATAAAAACTGA-TAAAATCTTTTGTTTTC-

>38_NC08_768082_770168_-_10/1-50

AAATT-ATTTCTGA-ATCAACTGTAAAAACGAA-TGAAACCCTTTGTTTTTC

>8_MC06_770761_772708_-_15/1-50

AAATT-ATTTCTGA-ATTAACTGTAAAAATGAA-CGAAACCTTTTGTTTTCC

>46_MC06_787716_789792_-_4/1-50

-TATTATTCACTGGTATTAAGTTGAAAAATAAG-TAAAAGCCTTTGTTTTTC

>22_Rhigh_267682_269737_+_10/1-50

ACATT--TCTCTGA-ATTTGTTATAAAAATTAG-TGAAACCCTTGATTTTC-

>51_CA06_441568_443908_+_9/1-50

-TAAAGATGTGCGGTTTTAACTATAAAAACTAA-TAAAACTCTTTGTTTTTA

>4_NC95_266035_268114_+_8/1-50

--ATTGAGTTGTTGTATTTAATATATAAAATAGCTAAAATCCTTTAAAAATC

>15_CA06_268203_270270_+_6/1-50

--ATTGAGTTGTTGTATTTAATATATAAAATAGCTAAAATCCTTTAAAAATC

>46_W101_774540_776592_-_4/1-50

-TATTATTCACTGGTATTAAGTTGAAAAATAAG-TAAAAGCCTTTGTTTTTC

>63_NC08_551762_553841_-_8/1-50

----TAAGTAGTTGTAAGTACGATAAAAACAAGGTAAAACCTTTTGTTTTCC

>30_MC06_541993_544096_-_9/1-50

AAATT-ATTTCTGA-ATCAACTGTAAAAACGAA-TGAAACCCTTTGTTTTTC

>38_Rhigh_818886_821002_-_18/1-50

-ACTATTTTTTTTGAATTAATGACAAAAAAC-GCTAAAACCTTTTATTTCTA

>32_Rhigh_265343_267347_+_14/1-50

TAATCTAGTTATGGTTTAAGATATAAAAACTTATAAAA-CCTTTTATTTTC-

>42_NC08_772916_774962_-_11/1-50

--ATTGAGTTGTTGTATCTAATATATAAAATAGCTAAAATCCTTTAAAAATC

>14_Rlow_595068_597117_-_11/1-50

ATAAGTTGTGATGGTTTTAACTATAAAAACTCA-CAAAACCTTTTGTTTTC-

>46_NY01_800390_802466_-_4/1-50

-TATTATTCACTGGTATTAAGTTGAAAAATAAG-TAAAAGCCTTTGTTTTTC

>15_VA94_284363_286460_+_6/1-50

--ATTGAGTTGTTGTATTTAATATATAAAATAGCTAAAATCCTTTAAAAATC

>6_NY01_281167_283186_+_8/1-50

--ATTGAGTTGTTGTATTTAATATATAAAATAGCTAAAATCCTTTAGAAATC

>25_Rhigh_838046_840170_-_6/1-50

-------CATTTTACATTTAGTCAAAAAACCGA-CGAAAGTCTTTGTTTATA

4. Alignment of 344 downstream flanks of non-12-GAA (GAA)n

>3_VA94_560912_562973_-_11/1-50

GTTCTTAGAAGTTTAGGGGTATGGGGTTGGGCAAGTGAGCGAAAATAAAC

>17_W101_536401_538531_-_6/1-50

GTTCTTAGAAGTTTAGGAGTTAGCGGATGCTCTGATCAGCGAAAATAAAC

>64_NC08_775261_777337_-_4/1-50

GATTTTAGAAGTTCTTAAGTTTGGGCCCCGTCTGATCAGCGAAAATAAAC

>23_Rlow_270092_270668_+_7/1-50

GTTCTTAGAAGTTTTAAG-------GCCGTATTTATGGGCGACAATTATG

>13_NC96_571466_573551_-_6/1-50

GTTCTTAGGAGTTCTGGGGTTTTCGTTTGGTCTGATCAGCGAAAATAAAC

>17_NC95_544696_546790_-_11/1-50

GTTCTTAGAAGTTTAGGAGTTAGCGGATGCTCTGATCAGCGAAAATAAAC

>48_W101_260641_262669_+_10/1-50

GTTCTTAGAAGTTTAGGAGTTAGCGACTACTCTGATCGGCGAAAATAAAC

>10_Rhigh_502397_504434_+_7/1-50

GTTCG-GGGAGTTCGGGGAGTTTGGTCTGACTTGATCTGCG---------

>27_Rlow_500056_502192_+_15/1-50

GTTCTTAGAAGTTTAGGGGTATGGGTTTGGGCAAGTGAGCGAAAATAAAC

>24_Rhigh_277083_279180_+_8/1-50

GTTCTTAGGAGTTCTGGGGTTTGGGAATCCTGTGATCAGCGAAAATTAAG

>15_CA06_446599_448672_+_10/1-50

GTTCTTAGAAGTTCTGTGGTCTGGGGTTGGTTTGATCAGCGAAAATAAAC

>4_Rlow_258271_260311_+_10/1-50

GTTCTTAGGAGTTCTGTGGTCTGGGGTTGGTTTGATCAGCGAAAATAAAC

>62_VA94_797027_799074_-_9/1-50

---CTTAGAAGTTTAGAGGTTTGGGGCTCGTCTGATCGACGAAAATAAAC

>14_NC08_547033_549127_-_6/1-50

GTTCTTAGAAGTTTAGGAGTTAGCGACTGCTCTGATCGGCGAAAATAAAT

>45_MC06_765767_768017_-_4/1-50

GATCTTAGAAGTTCTTAAGTTTGGGTCCCGTCTGATCGGTGAAAATTAAG

>34_Rhigh_483110_485333_+_10/1-50

GTTCTTAGAAGTTTAGGAGTTAGCGGATGCTCTGATCAGCGAAAATAAAC

>30_CA06_797230_799332_-_4/1-50

GTTCTTAGAAGTTCTGTGGTCTGGGGTTGGTTTGATCAGCGAAAATAAAC

>3_NC08_544680_546741_-_5/1-50

GTTCTTAGAAGTTTAGGGGTATGGGGTTGGGCAAGTGAGCGAAAATAAAC

>0_Rlow_253105_255361_+_8/1-50

GTTCTTAGGAGTTCTGGGGTTTGGGAATCCTGTGATCTGCGAAAATTAAG

>23_Rhigh_255818_256400_+_8/1-50

GTTCTTAGAAGTTTTAAG-------GCCGTATTTATGGGCGACAATTATG

>22_S6_806718_808674_-_4/1-50

GTTCTTAGGAGTTCTGGGGTTTGAGGATCGTTTGATCAGCGAAAATAAAC

>49_NC96_456843_458895_+_8/1-50

GTTCTTAGGAGTTCGGGGGTTTTCGTTTGGTCTGATCAGCGAAAATAAAC

>8_NY01_783167_785114_-_10/1-50

GTTCTTAGGAGTTCTGGGGTTTGGGGCTGGTTTGATCAGTGAAAATTAAG

>12_NC08_532333_534364_-_8/1-50

GTTCTTAGAAGTTTAGAGGTTTGGGGTTGGTCTGATCGGCGAAAATAAAC

>6_NC08_271015_273034_+_9/1-50

GTTCTTAGAAGTTTAGGAGTTAGCGGATGCTCTGATCAGCGAAAATAAAC

>27_NC96_263622_265692_+_13/1-50

GTTCTTAGGAGTTCTGGGGTTTGGGGCTGGTTTGATCAGTGAAAATTAAG

>14_Rhigh_594931_596980_-_7/1-50

GTTCTTAGAAGTTTAGGGGTATGGGGTTGGGCAAGTGAGCGAAAATAAAC

>47_W101_750416_752723_-_6/1-50

GTTCTTAGAAGTTTAGGAGTTAGCGACTACTCTGATCAGCGAAAATAAAT

>9_S6_808983_811068_-_10/1-50

GTTCTTAGGAGTTCGGGGAGTTTAGCCTGGCTCGATCGGCGAAAATTAAG

>13_NC96_268420_270511_+_8/1-50

GTTCTTAGGAGTTCTGGGGTTTGGGGCTGGTCTGATCGACGAAAATAAAC

>42_NC95_786317_788363_-_7/1-50

---CTTAGAAGTTTAGAGGTTTGGGGCTCGTCTGATCGACGAAAATAAAC

>3_NC95_781574_783635_-_6/1-50

GTTCTTAGAAGTTTAGGGGTATGGGGTTGGGCAAGTGAGCGAAAATAAAC

>45_NY01_778152_780420_-_4/1-50

GATCTTAGAAGTTCTTAAGTTTGGGTCCCGTCTGATCGGTGAAAATTAAG

>21_Rlow_604390_606655_-_13/1-50

GTTCTTAGAAGTTTTAAGTTTTCAGGCTGTTTTTATGGATGACAATTAAG

>45_CA06_789960_792210_-_5/1-50

GATCTTAGAAGTTCTTAAGTTTGGGTCCCGTCTGATCGGTGAAAATTAAG

>54_Rlow_739532_740123_-_5/1-50

GGCACAAGAAGAACAAGCTGAAGAAAATGTTGAAGCCACTCCAACTCA--

>50_S6_462439_464548_+_22/1-50

GTTCTTAGGAGTTCTGGGGTTTGGGGCTGGTTTGATCGGAGAAAATAAAC

>49_NY01_554651_556703_-_9/1-50

GTTCTTAGGAGTTCGGGGGTTTTCGTTTGGTCTGATCAGCGAAAATAAAC

>27_MC06_258651_260721_+_9/1-50

GTTCTTAGGAGTTATGGGGTTTGGGGCTGGTTTGATCAGTGAAAATTAAG

>52_MC06_556715_558794_-_11/1-50

GTTCTTAGAAGTTTTGGGGCTTTCGGCTGAACTAATCAGCGAAAATTAAG

>5_CA06_444268_446263_+_8/1-50

GTTCTTAGAAGTTTAGGAGTTAGCGGATGCTCTGATCAGCGAAAATAAAC

>2_MC06_271068_273087_+_13/1-50

GTTCTTAGAAGTTTAGGAGTTAGCGGATGCTCTGATCAGCGAAAATAAAC

>12_MC06_532352_534359_-_14/1-50

GTTCTTAGAAGTTTAGAGGTTTGGGGTTGGTCTGATCGGCGAAAATAAAC

>20_CA06_265397_267836_+_7/1-50

GATCTTAGAAGTTCTGGGT-TTTGGGCTTGCTGGATAGGTGAAAATAAAT

>4_MC06_261060_263139_+_10/1-50

GTTCTTAGAAGTTTAGGAGTTAGCGACTACTCTGATCGGCGAAAATAAAC

>50_NC96_454447_456541_+_8/1-50

GTTCTTAGAAGTTTAGGAGTTAGCGACTGCTCTGATCAGCGAAAATAAAT

>3_NY01_793317_795378_-_5/1-50

GTTCTTAGAAGTTTAGGGGTATGGGGTTGGGCAAGTGAGCGAAAATAAAC

>29_NC95_539880_541977_-_5/1-50

GTTCTTAGGAGTTCTGGGGTTTTCGTTTGGTCTGATCGGCGAAAATAAAC

>12_S6_567664_569698_-_10/1-50

GTTCTTAGGAGTTCTGGGGTTTTCGTTTGGT-TGATCGGCGAAAATAAAT

>6_Rlow_291870_293976_+_8/1-50

GTTCTTAGAAGTTTAGGAGTTAGCGGATGCTCTGATCAGCGAAAATAAAC

>12_F_502115_504104_+_13/1-50

GATCTTAGAAGTTCTTAAGTTTGGGTCTCGTCTGATCGGTGAAAATTAAG

>31_Rlow_607028_609215_-_10/1-50

GTTCTTAGGAGTTCTGGGGTTTGGGAATCCTGTGATCAGCGAAAATTAAG

>45_NC96_799285_801535_-_4/1-50

GATCTTAGAAGTTCTTAAGTTTGGGTCCCGTCTGATCGGTGAAAATTAAG

>13_VA94_279156_281247_+_10/1-50

GTTCTTAGGAGTTCTGGGGTTTGGGGCTGGTCTGATCGACGAAAATAAAC

>3_S6_264317_266366_+_16/1-50

GTTCTTAGGAGTTCTGGAGTTTTGAGCTGGTTTGATCGACGAAAATAAAC

>9_Rlow_828737_830864_-_9/1-50

GTTCTTAGAAGTTTAGGAGTGTTGGATTGGCCTAGTGAGCGAAAATAAAC

>26_W101_255829_257911_+_7/1-50

GTTCTTAGGAGTTCTGGGGTTTGGGAATCCTGTGATCTGCGAAAATTAAG

>13_NC95_542275_544360_-_14/1-50

GTTCTTAGGAGTTCTGGGGTTTTCGTTTGGTCTGATCAGCGAAAATAAAC

>12_NC95_537546_539553_-_10/1-50

GTTCTTAGAAGTTTAGAGGTTTGGGGTTGGTCTGATCGGCGAAAATAAAC

>24_NC08_763341_765438_-_19/1-50

GTTCTTAGGAGTTCTGGAGCTTTGGTTTGGCTTGATGAGCGAAAATAAAT

>9_CA06_799642_801799_-_9/1-50

GTTCTTAGAAGTTTAGGAGTTAGCGGATGCTCTGATCAGCGAAAATAAAC

>46_NY01_569002_571054_-_15/1-50

GTTCTTAGAAGTTTTGGGGCTTTCGGCTGAACTAATCAGCGAAAATTAAG

>29_CA06_563633_565730_-_8/1-50

GTTCTTAGGAGTTCTGGGGTTTTCGTTTGGTCTGATCGGCGAAAATAAAC

>42_NC08_549429_551475_-_7/1-50

---CTTAGAAGTTTAGAGGTTTGGGGCTCGTCTGATCGACGAAAATAAAC

>44_MC06_528257_529718_-_7/1-50

GTTCTTAAAAGTTCTGGGGTTTTCGTTTGGTCTGATCGGCGAAAATAAAC

>59_S6_801732_804117_-_6/1-50

GTTCTTAGAAGTTTAGTAGTGTGAGTCAACATTTTTAAGCGAAAATAATG

>46_NC95_788650_790726_-_4/1-50

GATTTTAGAAGTTCTTAAGTTTGGGCCCCGTCTGATCAGCGAAAATAAAC

>14_CA06_807474_809568_-_9/1-50

GTTCTTAGAAGTTTAGGAGTTAGCGACTGCTCTGATCGGCGAAAATAAAT

>2_Rhigh_495106_497206_+_7/1-50

ATTCTTAGGAGTTCTGGGGTTTGGGGTTGAGCTAATCAGCGAAAATAAAC

>35_Rlow_492855_494841_+_13/1-50

GTTCTTAGAAGTTCTGGAGTCTTGGTTTGGCTTGATGAGCGAAAATAAAC

>42_NC96_819194_821240_-_8/1-50

---CTTAGAAGTTTAGAGGTTTGGGGCTCGTCTGATCGACGAAAATAAAC

>32_Rlow_265429_267433_+_15/1-50

GATCTTAGAAGTTCTTAAGTTTGGGTCTCGTCTGATCGGTGAAAATTAAG

>26_NY01_261177_263259_+_11/1-50

GTTCTTAGGAGTTCTGGGGTTTGGGAATCCTGTGATCTGCGAAAATTAAG

>9_VA94_786841_788968_-_10/1-50

GTTCTTAGAAGTTTAGGAGTTAGCGGATGCTCTGATCAGCGAAAATAAAC

>45_NC08_760772_763028_-_4/1-50

GATCTTAGAAGTTCTTAAGTTTGGGTCCCGTCTGATCGGTGAAAATTAAG

>10_NC96_471163_473353_+_9/1-50

GTTCTTAGGAGTTCTGGGGTTTTCGTTTGGTCTGATCGGCGAAAATAAAC

>33_Rhigh_480559_482746_+_10/1-50

GTTCTTAGGAGTTCTGGGGTTTTCGTTTGGTCTGATCGGCGAAAATAAAC

>1_Rlow_260639_262661_+_11/1-50

GTTCTTAGAAGTTTTGGGGTTTGGGAATCCTGTGATCAGCGAAAATTAAG

>21_Rhigh_604235_606500_-_17/1-50

GTTCTTAGAAGTTTTAAGTTTTCAGGCTGTTTTTATGGATGACAATTAAG

>42_NC96_584055_586101_-_11/1-50

---CTTAGAAGTTTAGAGGTTTGGGGCTCGTCTGATCGACGAAAATAAAC

>27_W101_258232_260302_+_8/1-50

GTTCTTAGGAGTTCTGGGGTTTGGGGCTGGTTTGATCAGTGAAAATTAAG

>9_S6_459965_462074_+_11/1-50

GTTCTTAGGAGTTCTGGGGTTTAGGGCTGGTCTGATCGGCGAAAATAAAC

>39_CA06_456108_458109_+_16/1-50

GTTCTTAGAAGTTTAGAGGTTTGGGGCTCGTCTGATCGACGAAAATAAAC

>61_VA94_779726_781823_-_6/1-50

GTTCTTAGGAGTTCTGGAGCTTTGGTTTGGCTTGATGAGCGAAAATAAAT

>10_CA06_465755_467945_+_10/1-50

GTTCTTAGGAGTTCTGGGGTTTTCGTTTGGTCTGATCGGCGAAAATAAAC

>44_NC95_533448_534909_-_8/1-50

GTTCTTAGGAGTTCTGGGGTTTTCGTTTGGTCTGATCGGCGAAAATAAAC

>9_Rhigh_828531_830658_-_9/1-50

GTTCTTAGAAGTTTAGGAGTGTTGGATTGGCCTAGTGAGCGAAAATAAAC

>29_Rlow_597443_599537_-_10/1-50

GTTCTTAGGAGTTCTGGAGTTTTGGGCTGGTTTGATCGGCGAAAATTAAG

>39_Rhigh_821368_823313_-_9/1-50

ATTCTTAGGAGTTCTGGGGTTTGGGGCTGGTCTGATCGGCGAAAATAAAC

>15_S6_584223_586272_-_15/1-50

GTTCTTAGGAGTTCTGGGGTTTGGGAATCCTGTGATCAGCGAAAATTAAG

>22_Rhigh_830943_833016_-_7/1-50

----------GTTCTGTGGTCTGGGGTTGGTTTGATCAGCGAAAATAAAC

>13_NY01_273592_275683_+_6/1-50

GTTCTTAGAAGTTAGGGGAGTTTGGTCTGGCTTGATCTGCGAAAATAAAC

>4_Rlow_272459_274499_+_7/1-50

GTTCTTAGGAGTTCTGTGGTCTGGGGTTGGTTTGATCAGCGAAAATAAAC

>22_Rlow_831149_833216_-_7/1-50

----------GTTCTGTGGTCTGGGGTTGGTTTGATCAGCGAAAATAAAC

>4_VA94_276755_278834_+_11/1-50

GTTCTTAGAAGTTTAGGAGTTAGCGACTACTCTGATCGGCGAAAATAAAC

>4_NC96_266025_268104_+_8/1-50

GTTCTTAGAAGTTTAGGAGTTAGCGACTACTCTGATCGGCGAAAATAAAC

>12_NC96_566716_568723_-_16/1-50

GTTCTTAGAAGTTTAGAGGTTTGGGGTTGGTCTGATCGGCGAAAATAAAC

>47_CA06_787227_789636_-_10/1-50

GTTCTTAGAAGTTTAGGAGTTAGCGACTACTCTGATCAGCGAAAATAAAT

>5_Rhigh_833358_835329_-_13/1-50

GTTCTTAGGAGTTCTGTGGTCTGGGGTTGGTTTGATCAGCGAAAATAAAC

>4_S6_266678_268718_+_9/1-50

GTTCTTAGGAGTTCTGTGGTCTGGGGTTGGTTTGATCAGCGAAAATAAAC

>1_S6_261893_263936_+_4/1-50

GTTCTTAGGAGTTCTGGGGTTTGGGGCTGGTCTGATCGACGAAAATAAAC

>47_VA94_774526_776857_-_4/1-50

GTTCTTAGAAGTTTAGGAGTTAGCGACTACTCTGATCAGCGAAAATAAAT

>8_Rhigh_485674_487609_+_5/1-50

GTTCTTATGAGTTCTGGGGTTTGGGGCTGGTTTGATCAGTGAAAATTAAG

>44_W101_525147_526608_-_14/1-50

GTTCTTAGGAGTTCTGGGGTTTTCGTTTGGTCTGATCGGCGAAAATAAAC

>37_Rhigh_611077_611374_-_6/1-50

GTTCTTAGGAGTTCTATGGTCTGGGGTTGGTTTGATCAGCGAAAATAAAC

>20_Rhigh_286653_289041_+_7/1-50

GTTCTTAGAAGTTTAGAGGTTTGGGGCTCGTCTGTTCGACGAAAATAAAC

>13_VA94_553045_555130_-_11/1-50

GTTCTTAGGAGTTCTGGGGTTTTCGTTTGGTCTGATCAGCGAAAATAAAC

>29_Rhigh_597294_599388_-_8/1-50

GTTCTTAGGAGTTCTGGAGTTTTGGGCTGGTTTGATCGGCGAAAATTAAG

>22_Rhigh_281864_283925_+_9/1-50

GTTCTTAGAAGTTCTGTGGTCTGGGGTTGGTTTGATCAGCGAAAATAAAC

>33_NY01_545118_547296_-_9/1-50

---CTTAGAAGTTTAGAGGTTTGGGGCTCGTCTGATCGACGAAAATAAAC

>34_S6_579190_581509_-_13/1-50

GTTCTTAGAAGTTTAGTGGTATGGGGTTGGGCAAGTGAGCGAAAATTAAG

>38_Rlow_820753_821224_-_11/1-50

GTTCTTAGAAGTTTAGAGGTTTGGGACTCGTCTGATAGACGAAAATAAAC

>46_VA94_568030_570106_-_9/1-50

GTTCTTAGAAGTTTTGGGGCTTTCGGCTGAACTAATCAGCGAAAATTAAG

>30_NC95_773706_775809_-_5/1-50

GTTCTTAGAAGTTCTGTGGTCTGGGGTTGGTTTGATCAGCGAAAATAAAC

>17_F_499668_501783_+_9/1-50

GATCTTAGAAGTTCTTAAGTTTGGGTCTCGTCTGATCGGTGAAAATTAAG

>3_MC06_549600_551661_-_11/1-50

GTTCTTAGAAGTTTAGGGGTATGGGGTTGGGCAAGTGAGCGAAAATAAAC

>16_Rlow_490471_492517_+_9/1-50

GTTCTTAGGAGTTCTGGGGTTTGGGAATCCTGTGATCAGCGAAAATTAAG

>24_F_497265_499338_+_13/1-50

GTTTTTAGAAGTTTAGGGGTATGGAGTTGGGCAAGTGAGCGAAAATAAAC

>42_NY01_798057_800103_-_7/1-50

---CTTAGAAGTTTAGAGGTTTGGGGCTCGTCTGATCGACGAAAATAAAC

>6_W101_267695_269714_+_8/1-50

GTTCTTAGAAGTTTAGGAGTTAGCGGATGCTCTGATCAGCGAAAATAAAC

>3_CA06_805118_807179_-_6/1-50

GTTCTTAGAAGTTTAGGGGTATGGGGTTGGGCAAGTGAGCGAAAATAAAC

>20_NC96_270809_273248_+_6/1-50

GATCTTAGAAGTTCTGGGT-TTTGGGCTTGCTGGATAGGTGAAAATAAAT

>36_Rhigh_497529_499458_+_11/1-50

GTTATTAGAAGTTTAGGAGTTAGCGACTGCTCTGATCAGCGAAAATAAAT

>13_NY01_268402_270493_+_16/1-50

GTTCTTAGGAGTTCTGGGGTTTGGGGCTGGTCTGATCGACGAAAATAAAC

>17_NC08_539528_541628_-_16/1-50

GTTCTTAGAAGTTTAGGAGTTAGCGGATGCTCTGATCAGCGAAAATAAAC

>51_NC96_447141_449379_+_9/1-50

GTTCTTAGGAGTTCTGGGGTTTTCGTTTGGTCTGATCGGCGAAAATAAAC

>0_F_248320_250951_+_10/1-50

GTTCTTAGGAGTTCTGGGGTTTGGGAATCCTGTGATCTGCGAAAATTAAG

>46_VA94_799367_801443_-_4/1-50

GATTTTAGAAGTTCTTAAGTTTGGGCCCCGTCTGATCAGCGAAAATAAAC

>20_Rlow_286739_289127_+_7/1-50

GTTCTTAGAAGTTTAGAGGTTTGGGGCTCGTCTGTTCGACGAAAATAAAC

>39_NC96_461504_463505_+_11/1-50

GTTCTTAGAAGTTTAGAGGTTTGGGGCTCGTCTGATCGACGAAAATAAAC

>17_CA06_568458_570564_-_7/1-50

GTTCTTAGAAGTTTAGGAGTTAGCGGATGCTCTGATCAGCGAAAATAAAC

>16_NC96_273615_275724_+_6/1-50

GTTCTTAGAAGTTAGGGGAGTTTGGTCTGGCTTGATCTGCGAAAATAAAC

>15_S6_813835_815878_-_16/1-50

GTTCTTAGGAGTTCTGGGGTTTTCGTTTGGTCTGATCGGCGAAAATTAAG

>22_Rlow_267768_269823_+_10/1-50

GTTCTTAGAAGTTCTGTGGTCTGGGGTTGGTTTGATCAGCGAAAATAAAC

>13_CA06_263005_265096_+_6/1-50

GTTCTTAGGAGTTCTGGGGTTTGGGGCTGGTCTGATCGACGAAAATAAAC

>4_F_795960_797976_-_14/1-50

GTTCTTAGGAGTTCTGTGGTCTGGGGTTGGTTTGATCAGCGAAAATAAAC

>45_VA94_777163_779413_-_4/1-50

GATCTTAGAAGTTCTTAAGTTTGGGTCCCGTCTGATCGGTGAAAATTAAG

>6_CA06_270567_272586_+_9/1-50

GTTCTTAGAAGTTTAGGAGTTAGCGGATGCTCTGATCAGCGAAAATAAAC

>29_NC96_569068_571165_-_6/1-50

GTTCTTAGGAGTTCTGGGGTTTTCGTTTGGTCTGATCGGCGAAAATAAAC

>23_S6_812654_813461_-_19/1-50

GTTCTTAGAAGTTTAGGAGTTAGTGACTGCTCTGATCAGCGAAA------

>33_Rlow_480651_482838_+_9/1-50

GTTCTTAGGAGTTCTGGGGTTTTCGTTTGGTCTGATCGGCGAAAATAAAC

>32_Rhigh_279528_281532_+_15/1-50

GATCTTAGAAGTTCTTAAGTTTGGGTCTCGTCTGATCGGTGAAAATTAAG

>25_Rlow_838234_840358_-_7/1-50

GTTCTTAGAAGTTCAGGAGTGTCGGGTTGAGCTAGTGAGCGAAAATTAAG

>6_VA94_286763_288782_+_11/1-50

GTTCTTAGAAGTTTAGGAGTTAGCGGATGCTCTGATCAGCGAAAATAAAC

>13_NC95_268424_270515_+_6/1-50

GTTCTTAGGAGTTCTGGGGTTTGGGGCTGGTCTGATCGACGAAAATAAAC

>13_NC08_537107_539192_-_14/1-50

GTTCTTAGGAGTTCTGGGGTTTTCGTTTGGTCTGATCAGCGAAAATAAAC

>12_NY01_542729_544739_-_15/1-50

GTTCTTAGAAGTTTTAAGTTTTCAGGCTGTTTTTATGGATGACAATTAAG

>13_CA06_566037_568122_-_14/1-50

GTTCTTAGGAGTTCTGGGGTTTTCGTTTGGTCTGATCAGCGAAAATAAAC

>39_Rlow_821569_823510_-_8/1-50

ATTCTTAGGAGTTCTGGGGTTTGGGGCTGGTCTGATCGGCGAAAATAAAC

>4_W101_543944_545993_-_10/1-50

GTTCTTAGAAGTTTTGGGGCTTTCGGCTGAACTAATCAGCGAAAATTAAG

>52_W101_767449_769519_-_10/1-50

GTTCTTAGAAGTTCTGTGGTCTGGGGTTGGTTTGATCAGCGAAAATAAAC

>13_S6_581827_583894_-_10/1-50

GTTCTTAGAAGTTCTGGAGTCTTGGTTTGGCTTGATGAGCGAAAATAAAC

>23_Rlow_255877_256471_+_10/1-50

GTTCTTAGAAGTTTTAAG-------GCCGTATTTATGGGCGACAATTATG

>15_Rhigh_289417_291490_+_8/1-50

GTTCTTAGAAGTTAGGGGAGTTTGGTCTGGCTTGATCTGCGAAAATAAAC

>40_Rhigh_826006_828211_-_9/1-50

GTTCTTAGAAGTTTAGAGGTTTGGGGCTCGTCTGATCGACGAAAATAAAC

>47_NC08_758243_760466_-_4/1-50

GTTCTTAGAAGTTTAGGAGTTAGCGACTACTCTGATCAGCGAAAATAAAT

>5_NY01_561871_563866_-_9/1-50

GTTCTTAGAAGTTTAGGAGTTAGCGGATGCTCTGATCAGCGAAAATAAAC

>44_S6_562996_565003_-_16/1-50

GTTCTTAGGAGTTCTGGGGTTTTCGTTTGGTCTGATCGGCGAAAATAAAC

>26_CA06_255804_257886_+_4/1-50

GTTCTTAGGAGTTCTGGGGTTTGGGAATCCTGTGATCTGCGAAAATTAAG

>17_Rlow_488036_490148_+_11/1-50

GTTCTTAGAAGTTCTGTGGTCTGGGGTTGGTTTGATCAGCGAAAATAAAC

>50_NY01_557008_559102_-_18/1-50

GTTCTTAGAAGTTTAGGAGTTAGCGACTGCTCTGATCAGCGAAAATAAAT

>9_NC96_808984_811111_-_11/1-50

GTTCTTAGAAGTTTAGGAGTTAGCGGATGCTCTGATCAGCGAAAATAAAC

>47_NC96_796558_798979_-_4/1-50

GTTCTTAGAAGTTTAGGAGTTAGCGACTACTCTGATCAGCGAAAATAAAT

>14_NC95_552480_554574_-_9/1-50

GTTCTTAGAAGTTTAGGAGTTAGCGACTGCTCTGATCGGCGAAAATAAAT

>42_NC95_554885_556931_-_7/1-50

---CTTAGAAGTTTAGAGGTTTGGGGCTCGTCTGATCGACGAAAATAAAC

>9_MC06_775500_777585_-_5/1-50

GTTCTTAGAAGTTTAGGAGTTAGCGGATGCTCTGATCAGCGAAAATAAAC

>36_CA06_463384_465280_+_10/1-50

GTTCTTAGAAGTTTTAAGTTTTCAGGCTGTTTTTATGGATGACAATTAAG

>17_S6_586613_588743_-_11/1-50

GTTCTTAGGAGTTCTGGGGTTTTCGTTTGGTCTGATCGGCGAAAATAAAC

>44_NY01_538634_540095_-_7/1-50

GTTCTTAGGAGTTCTGGGGTTTTCGTTTGGTCTGATCGGCGAAAATAAAC

>26_F_253707_255777_+_8/1-50

GTTCTTAGGAGTTTAGGAATGTTGGATTGGCCTAGTGAGCGAAAATAAAC

>1_Rlow_274827_276849_+_11/1-50

GTTCTTAGAAGTTTTGGGGTTTGGGAATCCTGTGATCAGCGAAAATTAAG

>23_MC06_268674_270759_+_8/1-50

GTTCTTAGAAGTTAGGGGAGTTTGGTCTGGCTTGATCTGCGAAAATAAAC

>24_NY01_780733_782830_-_11/1-50

GTTCTTAGGAGTTCTGGAGCTTTGGTTTGGCTTGATGAGCGAAAATAAAT

>6_Rhigh_291778_293884_+_6/1-50

GTTCTTAGAAGTTTAGGAGTTAGCGGATGCTCTGATCAGCGAAAATAAAC

>15_NC95_273625_275710_+_6/1-50

GTTCTTAGAAGTTAGGGGAGTTTGGTCTGGCTTGATCTGCGAAAATAAAC

>12_VA94_548313_550320_-_9/1-50

GTTCTTAGAAGTTTAGAGGTTTGGGGTTGGTCTGATCGGCGAAAATAAAC

>22_Rlow_281938_284005_+_9/1-50

GTTCTTAGAAGTTCTGTGGTCTGGGGTTGGTTTGATCAGCGAAAATAAAC

>20_VA94_281557_283996_+_10/1-50

GATCTTAGAAGTTCTGGGT-TTTGGGCTTGCTGGATAGGTGAAAATAAAT

>30_NY01_785456_787559_-_5/1-50

GTTCTTAGAAGTTCTGTGGTCTGGGGTTGGTTTGATCAGCGAAAATAAAC

>26_NC95_261229_263311_+_6/1-50

GTTCTTAGGAGTTCTGGGGTTTGGGAATCCTGTGATCTGCGAAAATTAAG

>6_S6_271453_273514_+_22/1-50

GTTCTTAGAAGTTCTGTGGTCTGGGGTTGGTTTGATCAGCGAAAATAAAC

>1_Rhigh_274747_276769_+_9/1-50

GTTCTTAGAAGTTTTGGGGTTTGGGAATCCTGTGATCAGCGAAAATTAAG

>0_CA06_802173_804810_-_8/1-50

GTTCTTAGGAGTTCTGTGGTCTGGGGTTGGTTTGATCAGCGAAAATAAAC

>20_MC06_265862_268301_+_16/1-50

GATCTTAGAAGTTCTGGGT-TTTGGGCTTGCTGGATAGGTGAAAATAAAT

>26_VA94_271916_273998_+_10/1-50

GTTCTTAGGAGTTCTGGGGTTTGGGAATCCTGTGATCTGCGAAAATTAAG

>3_NC96_579306_581367_-_8/1-50

GTTCTTAGAAGTTTAGGGGTATGGGGTTGGGCAAGTGAGCGAAAATAAAC

>47_S6_798710_800825_-_11/1-50

GTTCTTAGAAGTTTAGAGGTTTGGGACTCGTCTGATAGACGAAAATAAAC

>42_MC06_785368_787414_-_9/1-50

---CTTAAAAGTTTAGAGGTTTGGGGCTCGTCTGATCGACGAAAATAAAC

>42_VA94_565700_567746_-_6/1-50

---CTTAGAAGTTTAGAGGTTTGGGGCTCGTCTGATCGACGAAAATAAAC

>42_CA06_578362_580408_-_13/1-50

---CTTAGAAGTTTAGAGGTTTGGGGCTCGTCTGATCGACGAAAATAAAC

>4_CA06_260616_262695_+_9/1-50

GTTCTTAGAAGTTTAGGAGTTAGCGACTACTCTGATCGGCGAAAATAAAC

>24_NC95_768977_771074_-_13/1-50

GTTCTTAGGAGTTCTGGAGCTTTGGTTTGGCTTGATGAGCGAAAATAAAT

>8_S6_804441_806394_-_14/1-50

GTTCTTAGGAGTTCTGGGGTTTTGGTTTGGCTTGATGAGCGAAAATAAAC

>0_Rhigh_253052_255308_+_8/1-50

GTTCTTAGGAGTTCTGGGGTTTGGGAATCCTGTGATCTGCGAAAATTAAG

>47_NC95_763720_766105_-_4/1-50

GTTCTTAGAAGTTTAGGAGTTAGCGACTACTCTGATCAGCGAAAATAAAT

>24_Rhigh_262901_264998_+_8/1-50

GTTCTTAGGAGTTCTGGGGTTTGGGAATCCTGTGATCAGCGAAAATTAAG

>24_CA06_792526_794623_-_8/1-50

GTTTTTAGGAGTTCTGGAGCTTTGGTTTGGCTTGATGAGCGAAAATAAAT

>17_VA94_555457_557575_-_11/1-50

GTTCTTAGAAGTTTAGGAGTTAGCGGATGCTCTGATCAGCGAAAATAAAC

>3_MC06_780598_782659_-_9/1-50

GTTCTTAGAAGTTTAGGGGTATGGGGTTGGGCAAGTGAGCGAAAATAAAC

>3_NC96_814427_816488_-_6/1-50

GTTCTTAGAAGTTTAGGGGTATGGGGTTGGGCAAGTGAGCGAAAATAAAC

>42_W101_772204_774250_-_8/1-50

---CTTAGAAGTTTAGAGGTTTGGGGCTCGTCTGATCGACGAAAATAAAC

>4_NC08_261052_263131_+_8/1-50

GTTCTTAGAAGTTTAGGAGTTAGCGACTACTCTGATCGGCGAAAATAAAC

>55_NY01_559440_561525_-_13/1-50

GTTCTTAGAAGTTCTGTGGTCTGGGGTTGGTTTGATCAGCGAAAATAAAC

>0_MC06_546943_549289_-_9/1-50

GTTCTTAGGAGTTCTGTGGTCTGGGGTTGGTTTGATCAGCGAAAATAAAC

>46_NC96_821530_823606_-_4/1-50

GATTTTAGAAGTTCTTAAGTTTGGGCCCCGTCTGATCAGCGAAAATAAAC

>30_VA94_784431_786516_-_9/1-50

GTTCTTAGAAGTTCTGTGGTCTGGGGTTGGTTTGATCAGCGAAAATAAAC

>12_Rhigh_592634_594635_-_10/1-50

----------GTTTAGGAGTGTCGGGTTGAGCTAGTGAGCGAAAATAAAC

>48_NY01_552318_554346_-_9/1-50

GTTCTTAGAAGTTTAGAGGTTTGGGACTCGTCTGATCGACGAAAATAAAC

>8_NC08_765799_767746_-_8/1-50

GTTCTTAGGAGTTCTGGGGTTTGGGGCTGGTTTGATCAGTGAAAATTAAG

>5_NC96_449732_451727_+_6/1-50

GTTCTTAGAAGTTTAGGAGTTAGCGGATGCTCTGATCAGCGAAAATAAAC

>34_CA06_458414_460544_+_10/1-50

GTTCTTAGAAGTTTAGGAGTTAGCGACTGCTCTGATCAGCGAAAATAAAT

>13_W101_533995_536080_-_9/1-50

GTTCTTAGGAGTTCTGGGGTTTTCGTTTGGTCTGATCAGCGAAAATAAAC

>24_Rlow_262984_265081_+_11/1-50

GTTCTTAGGAGTTCTGGGGTTTGGGAATCCTGTGATCAGCGAAAATTAAG

>22_F_251355_253422_+_19/1-50

GTTCTTAGAAGTTCTGTGGTCTGGGGTTGGTTTGATCAGCGAAAATAAAC

>44_NC96_562609_564070_-_11/1-50

GTTCTTAGGAGTTCTGGGGTTTTCGTTTGGTCTGATCGGCGAAAATAAAC

>4_Rhigh_272385_274425_+_8/1-50

GTTCTTAGGAGTTCTGTGGTCTGGGGTTGGTTTGATCAGCGAAAATAAAC

>24_Rlow_277172_279269_+_11/1-50

GTTCTTAGGAGTTCTGGGGTTTGGGAATCCTGTGATCAGCGAAAATTAAG

>44_CA06_557210_558671_-_7/1-50

GTTCTTAGGAGTTCTGGGGTTTTCGTTTGGTCTGATCGGCGAAAATAAAC

>3_W101_541559_543620_-_10/1-50

GTTCTTAGAAGTTTAGGGGTATGGGGTTGGGCAAGTGAGCGAAAATAAAC

>20_NY01_270788_273227_+_5/1-50

GATCTTAGAAGTTCTGGGT-TTTGGGCTTGCTGGATAGGTGAAAATAAAT

>34_Rlow_483196_485419_+_8/1-50

GTTCTTAGAAGTTTAGGAGTTAGCGGATGCTCTGATCAGCGAAAATAAAC

>0_MC06_777947_780290_-_8/1-50

GTTCTTAGGAGTTCTGTGGTCTGGGGTTGGTTTGATCAGCGAAAATAAAC

>47_NY01_775551_777846_-_4/1-50

GTTCTTAGAAGTTTAGGAGTTAGCGACTACTCTGATCAGCGAAAATAAAT

>6_NC95_276007_278026_+_9/1-50

GTTCTTAGAAGTTTAGGAGTTAGCGGATGCTCTGATCAGCGAAAATAAAC

>10_Rlow_502531_504568_+_7/1-50

GTTCG-GGGAGTTCGGGGAGTTTGGTCTGACTTGATCTGCG---------

>26_NC08_256246_258328_+_8/1-50

GTTCTTAGGAGTTCTGGGGTTTGGGAATCCTGTGATCTGCGAAAATTAAG

>3_VA94_792281_794342_-_7/1-50

GTTCTTAGAAGTTTAGGGGTATGGGGTTGGGCAAGTGAGCGAAAATAAAC

>30_W101_760339_762443_-_10/1-50

GTTCTTAGAAGTTCTGTGGTCTGGGGTTGGTTTGATCAGCGAAAATAAAC

>20_NY01_275988_278427_+_9/1-50

GATCTTAGAAGTTCTGGGT-TTTGGGCTTGCTGGATAGGTGAAAATAAAT

>46_S6_259528_261616_+_9/1-50

GTTCTTAGAAGTTCTGTGGTCTGGGGTTGGTTTGATCAGCGAAAATAAAC

>29_VA94_550644_552741_-_7/1-50

GTTCTTAGGAGTTCTGGGGTTTTCGTTTGGTCTGATCGGCGAAAATAAAC

>3_CA06_573598_575659_-_6/1-50

GTTCTTAGAAGTTTAGGGGTATGGGGTTGGGCAAGTGAGCGAAAATAAAC

>38_CA06_809879_811925_-_13/1-50

---CTTAGAAGTTTAGAGGTTTGGGGCTCGTCTGATCGACGAAAATAAAC

>15_Rlow_289503_291576_+_8/1-50

GTTCTTAGAAGTTAGGGGAGTTTGGTCTGGCTTGATCTGCGAAAATAAAC

>5_S6_269082_271062_+_23/1-50

GTTCTTAGAAGTTTTGGGGTTTGGGAATCCTGTGATCAGCGAAAATTAAG

>29_NC08_534685_536782_-_14/1-50

GTTCTTAGGAGTTCTGGGGTTTTCGTTTGGTCTGATCGGCGAAAATAAAC

>27_NC95_263632_265702_+_8/1-50

GTTCTTAGGAGTTCTGGGGTTTGGGGCTGGTTTGATCAGTGAAAATTAAG

>0_VA94_789345_791982_-_5/1-50

GTTCTTAGGAGTTCTGTGGTCTGGGGTTGGTTTGATCAGCGAAAATAAAC

>56_NY01_564228_566340_-_14/1-50

GTTCTTAGAAGTTTAGGAGTTAGCGACTGCTCTGATCGGCGAAAATAAAT

>37_Rlow_611232_611529_-_7/1-50

GTTCTTAGGAGTTCTATGGTCTGGGGTTGGTTTGATCAGCGAAAATAAAC

>48_CA06_453786_455814_+_17/1-50

GTTCTTAGAAGTTTAGAGGTTTGGGACTCGTCTGATCGACGAAAATAAAC

>40_Rlow_826200_828405_-_13/1-50

GTTCTTAGAAGTTTAGAGGTTTGGGGCTCGTCTGATCGACGAAAATAAAC

>45_MC06_534698_536942_-_4/1-50

GATCTTAGAAGTTCTTAAGTTTGGGTCCCGTCTGATCGGTGAAAATTAAG

>46_NC96_586400_588476_-_10/1-50

GTTCTTAGAAGTTTTGGGGCTTTCGGCTGAACTAATCAGCGAAAATTAAG

>26_MC06_256245_258327_+_9/1-50

GTTCTTAGGAGTTCTGGGGTTTGGGAATCCTGTGATCTGCGAAAATTAAG

>15_NC96_452067_454140_+_11/1-50

GTTCTTAGAAGTTCTGTGGTCTGGGGTTGGTTTGATCAGCGAAAATAAAC

>5_Rlow_833558_835529_-_9/1-50

GTTCTTAGGAGTTCTGTGGTCTGGGGTTGGTTTGATCAGCGAAAATAAAC

>32_Rlow_279602_281606_+_10/1-50

GATCTTAGAAGTTCTTAAGTTTGGGTCTCGTCTGATCGGTGAAAATTAAG

>36_NC96_468795_470691_+_7/1-50

GTTCTTAGAAGTTTTAAGTTTTCAGGCTGTTTTTATGGATGACAATTAAG

>3_F_798303_800352_-_9/1-50

GTTCTTAGGAGTTCTGGAGTTTTGAGCTGGTTTGATCGACGAAAATAAAC

>46_F_805439_807527_-_4/1-50

GATCTTAGAAGTTCTTAAGTTTGGGCCCCGTCTGATCGGTGAAAATTAAG

>14_CA06_575954_578048_-_10/1-50

GTTCTTAGAAGTTTAGGAGTTAGCGACTGCTCTGATCGGCGAAAATAAAT

>14_W101_762771_764808_-_8/1-50

GTTCTTAGAAGTTTAGGAGTTAGCGACTGCTCTGATCGGCGAAAATAAAT

>30_S6_455211_457326_+_17/1-50

GTTCTTAGGAGTTCTGGGGTCTGGGGATCGTTTGATCAGCGAAAATAAAC

>44_VA94_544218_545679_-_7/1-50

GTTCTTAGGAGTTCTGGGGTTTTCGTTTGGTCTGATCGGCGAAAATAAAC

>8_CA06_794951_796898_-_10/1-50

----------GTTCTGGGGTTTGGGGCTGGTTTGATCAGTGAAAATTAAG

>43_NY01_550041_552042_-_10/1-50

GTTCTTAGAAGTTTAGAGGTTTGGGGCTCGTCTGATCGACGAAAATAAAC

>26_Rlow_284290_286423_+_9/1-50

GTTCTTAGAAGTTTAGGAGTGTTGGATTGGCCTAGTGAGCGAAAATAAAC

>34_NY01_547585_549733_-_11/1-50

GTTCTTAGAAGTTTAGGAGTTAGCGACTGCTCTGATCAGCGAAAATAAAT

>13_MC06_263443_265534_+_4/1-50

GTTCTTAGGAGTTCTGGGGTTTGGGGCTGGTCTGATCGACGAAAATAAAC

>3_NC95_550115_552176_-_9/1-50

GTTCTTAGAAGTTTAGGGGTATGGGGTTGGGCAAGTGAGCGAAAATAAAC

>49_CA06_451405_453457_+_8/1-50

GTTCTTAGGAGTTCGGGGGTTTTCGTTTGGTCTGATCAGCGAAAATAAAC

>8_NC95_771417_773364_-_10/1-50

GTTCTTAGGAGTTCTGGGGTTTGGGGCTGGTTTGATCAGTGAAAATTAAG

>8_Rlow_485760_487695_+_5/1-50

GTTCTTATGAGTTCTGGGGTTTGGGGCTGGTTTGATCAGTGAAAATTAAG

>9_NC95_776122_778249_-_11/1-50

GTTCTTAGAAGTTTAGGAGTTAGCGGATGCTCTGATCAGCGAAAATAAAC

>20_NC08_265839_268278_+_8/1-50

GATCTTAGAAGTTCTGGGT-TTTGGGCTTGCTGGATAGGTGAAAATAAAT

>12_Rlow_592768_594769_-_11/1-50

----------GTTTAGGAGTGTCGGGTTGAGCTAGTGAGCGAAAATAAAC

>44_NC08_528226_529687_-_11/1-50

GTTCTTAGGAGTTCTGGGGTTTTCGTTTGGTCTGATCGGCGAAAATAAAC

>26_NC96_261204_263286_+_9/1-50

GTTCTTAGGAGTTCTGGGGTTTGGGAATCCTGTGATCTGCGAAAATTAAG

>27_Rhigh_499922_502058_+_9/1-50

GTTCTTAGAAGTTTAGGGGTATGGGTTTGGGCAAGTGAGCGAAAATAAAC

>47_MC06_763220_765461_-_4/1-50

GTTCTTAGAAGTTTAGGAGTTAGCGACTACTCTGATCAGCGAAAATAAAT

>45_W101_753035_755309_-_5/1-50

GATCTTAGAAGTTCTTAAGTTTGGGTCCCGTCTGATCGGTGAAAATTAAG

>1_F_800711_802754_-_9/1-50

GTTCTTAGGAGTTCTGGGGTTTGGGGCTGGTCTGATCGACGAAA------

>34_W101_265262_267401_+_8/1-50

GTTCTTAGAAGTTTAGGAGTTAGCGACTGCTCTGATCAGCGAAAATAAAT

>6_F_791276_793289_-_13/1-50

GTTCTTAGAAGTTCTGTGGTCTGGGGTTGGTTTGATCAGCGAAAATAAAC

>42_S6_589038_591120_-_18/1-50

GTTTTTAGAAGTTTTGGGGCTTTCGGCTGAACTAATCAGCGAAAATTAAG

>58_S6_569986_572158_-_18/1-50

GTTCTTAGGAGTTCTGGGGTTTTCGTTTGGTCTGATCGGGGAAAATAAAC

>0_NC08_542023_544366_-_10/1-50

GTTCTTAGGAGTTCTGTGGTCTGGGGTTGGTTTGATCAGCGAAAATAAAC

>10_S6_464867_466910_+_14/1-50

GTTCTTAGGAGTTCGGGGGTTTTCGTTTGGTCTGATCAGCGAAAATAAAC

>4_NY01_265995_268062_+_8/1-50

GTTCTTAGAAGTTTAGGAGTTAGCGACTACTCTGATCGGCGAAAATAAAC

>4_Rhigh_258200_260240_+_10/1-50

GTTCTTAGGAGTTCTGTGGTCTGGGGTTGGTTTGATCAGCGAAAATAAAC

>30_MC06_773065_775168_-_8/1-50

GTTCTTAGAAGTTCTGTGGTCTGGGGTTGGTTTGATCAGC----------

>16_Rhigh_490409_492455_+_20/1-50

GTTCTTAGGAGTTCTGGGGTTTGGGAATCCTGTGATCAGCGAAAATTAAG

>26_Rhigh_284210_286337_+_9/1-50

GTTCTTAGAAGTTTAGGAGTGTTGGATTGGCCTAGTGAGCGAAAATAAAC

>42_MC06_554358_556404_-_15/1-50

---CTTAGAAGTTTAGAGGTTTGGGGCTCGTCTGATCGACGAAAATAAAC

>43_W101_263010_264957_+_11/1-50

GTTCTTAGAAGTTTAGAGGTTTGGGGCTCGTCTGATCGACGAAAATAAAC

>35_Rhigh_492796_494782_+_14/1-50

GTTCTTAGAAGTTCTGGAGTCTTGGTTTGGCTTGATGAGCGAAAATAAAC

>46_NC95_557218_559294_-_11/1-50

GTTCTTAGAAGTTTTGGGGCTTTCGGCTGAACTAATCAGCGAAAATTAAG

>17_NC96_573863_575969_-_11/1-50

GTTCTTAGAAGTTTAGGAGTTAGCGGATGCTCTGATCAGCGAAAATAAAC

>8_MC06_539692_541639_-_14/1-50

GTTCTTAGGAGTTCTGGGGTTTGGGGCTGGTTTGATCAGTGAAAATTAAG

>17_Rhigh_487941_490053_+_8/1-50

GTTCTTAGAAGTTCTGTGGTCTGGGGTTGGTTTGATCAGCGAAAATAAAC

>31_Rhigh_606885_609072_-_6/1-50

GTTCTTAGGAGTTCTGGGGTTTGGGAATCCTGTGATCAGCGAAAATTAAG

>0_NC95_778629_781266_-_8/1-50

GTTCTTAGGAGTTCTGTGGTCTGGGGTTGGTTTGATCAGCGAAAATAAAC

>24_MC06_768330_770427_-_10/1-50

GTTCTTAGGAGTTCTGGAGCTTTGGTTTGGCTTGATGAGCGAAAATAAAT

>8_NC96_804273_806220_-_8/1-50

GTTCTTAGGAGTTCTGGGGTTTGGGGCTGGTTTGATCAGTGAAAATTAAG

>0_NC95_547170_549807_-_8/1-50

GTTCTTAGGAGTTCTGTGGTCTGGGGTTGGTTTGATCAGCGAAAATAAAC

>8_VA94_782145_784092_-_9/1-50

GTTCTTAGGAGTTCTGGGGTTTGGGGCTGGTTTGATCAGTGAAAATTAAG

>14_NC96_816783_818877_-_11/1-50

GTTCTTAGAAGTTTAGGAGTTAGCGACTGCTCTGATCGGCGAAAATAAAT

>12_CA06_561305_563312_-_8/1-50

GTTCTTAGAAGTTTAGAGGTTTGGGGTTGGTCTGATCGGCGAAAATAAAC

>15_NY01_278800_280873_+_8/1-50

GTTCTTAGAAGTTAGGGGAGTTTGGTCTGGCTTGATCTGCGAAAATAAAC

>2_Rlow_495168_497274_+_8/1-50

ATTCTTAGGAGTTCTGGGGTTTGGGGTTGAGCTAATCAGCGAAAATAAAC

>0_S6_256524_259155_+_8/1-50

GTTCTTAGGAGTTCTGGGGTTTGGGAATCCTGTGATCTGCGAAAATTAAG

>50_CA06_449009_451103_+_18/1-50

GTTCTTAGAAGTTTAGGAGTTAGCGACTGCTCTGATCAGCGAAAATAAAT

>6_NC96_276045_278064_+_17/1-50

GTTCTTAGAAGTTTAGGAGTTAGCGGATGCTCTGATCAGCGAAAATAAAC

>46_CA06_812230_814282_-_4/1-50

GATTTTAGAAGTTCTTAAGTTTGGGCCCCGTCTGATCAGCAAAAATAAAC

>30_NC96_806556_808659_-_9/1-50

GTTCTTAGAAGTTCTGTGGTCTGGGGTTGGTTTGATCAGCGAAAATAAAC

>42_W101_765116_767162_-_7/1-50

---CTTAGAAGTTTAGAGGTTTGGGGCTCGTCTGATCGACGAAAATAAAC

>27_VA94_274343_276413_+_16/1-50

GTTCTTAGGAGTTCTGGGGTTTGGGGCTGGTTTGATCAGTGAAAATTAAG

>24_W101_755625_757722_-_8/1-50

GTTCTTAGGAGTTCTGGAGCTTTGGTTTGGCTTGATGAGCGAAAATAAAT

>23_Rhigh_270003_270591_+_6/1-50

GTTCTTAGAAGTTTTAAG-------GCCGTATTTATGGGCGACAATTATG

>60_S6_816229_818317_-_7/1-50

GTTCTTAGAAGTTCAGGAGTGTCGGGTTGAGCTAGTGAGCGAAAATTAAG

>0_NC96_811491_814128_-_5/1-50

GTTCTTAGGAGTTCTGTGGTCTGGGGTTGGTTTGATCAGCGAAAATAAAC

>13_NC08_263444_265535_+_7/1-50

GTTCTTAGGAGTTCTGGGGTTTGGGGCTGGTCTGATCGACGAAAATAAAC

>0_CA06_570932_573275_-_13/1-50

GTTCTTAGGAGTTCTGTGGTCTGGGGTTGGTTTGATCAGCGAAAATAAAC

>42_NY01_566666_568712_-_8/1-50

---CTTAGAAGTTTAGAGGTTTGGGGCTCGTCTGATCGACGAAAATAAAC

>14_VA94_563283_565377_-_13/1-50

GTTCTTAGAAGTTTAGGAGTTAGCGACTGCTCTGATCGGCGAAAATAAAT

>46_CA06_580713_582789_-_6/1-50

GTTCTTAGAAGTTTTGGGGCTTTCGGCTGAACTAATCAGCGAAAATTAAG

>24_NC96_801848_803945_-_8/1-50

GTTCTTAGGAGTTCTGGAGCTTTGGTTTGGCTTGATGAGCGAAAATAAAT

>1_Rhigh_260565_262587_+_10/1-50

GTTCTTAGAAGTTTTGGGGTTTGGGAATCCTGTGATCAGCGAAAATTAAG

>8_W101_758050_759997_-_10/1-50

GTTCTTAGGAGTTCTGGGGTTTGGGGCTGGTTTGATCAGTGAAAATTAAG

>27_CA06_258210_260280_+_9/1-50

GTTCTTAGGAGTTCTGGGGTTTGGGGCTGGTTTGATCAGTGAAAATTAAG

>14_MC06_782963_785057_-_9/1-50

GTTCTTAGAAGTTTAGGAGTTAGCGACTGCTCTGATCGGCGAAAATAAAT

>36_Rlow_497645_499574_+_27/1-50

GTTATTAGAAGTTTAGGAGTTAGCGACTGCTCTGATCAGCGAAAATAAAT

>27_NC08_258649_260719_+_8/1-50

GTTCTTAGGAGTTCTGGGGTTTGGGGCTGGTTTGATCAGTGAAAATTAAG

>20_NC95_270819_273258_+_8/1-50

GATCTTAGAAGTTCTGGGT-TTTGGGCTTGCTGGATAGGTGAAAATAAAT

>48_NC96_459197_461225_+_8/1-50

GTTCTTAGAAGTTTAGAGGTTTGGGACTCGTCTGATCGACGAAAATAAAC

>38_NC08_768082_770168_-_10/1-50

GTTCTTAGAAGTTCTGTGGTCTGGGGTTGGTTTGATCAGCGAAAATAAAC

>8_MC06_770761_772708_-_15/1-50

GTTCTTAGGAGTTCTGGGGTTTGGGGCTGGTTTGATCAGTGAAAATTAAG

>46_MC06_787716_789792_-_4/1-50

GATTTTAGAAGTTCTTAAGTTTGGGCCCCGTCTGATCAGCGAAAATAAAC

>22_Rhigh_267682_269737_+_10/1-50

GTTCTTAGAAGTTCTGTGGTCTGGGGTTGGTTTGATCAGCGAAAATAAAC

>51_CA06_441568_443908_+_9/1-50

GTTCTTAGGAGTTCTGGGGTTTTCGTTTGGTCTGATCGGCGAAAATAAAC

>4_NC95_266035_268114_+_8/1-50

GTTCTTAGAAGTTTAGGAGTTAGCGACTACTCTGATCGGCGAAAATAAAC

>15_CA06_268203_270270_+_6/1-50

GTTCTTAGAAGTTAGGGGAGTTTGGTCTGGCTTGATCTGCGAAAATAAAC

>46_W101_774540_776592_-_4/1-50

GATTTTAGAAGTTCTTAAGTTTGGGCCCCGTCTGATCAGCGAAAATAAAC

>63_NC08_551762_553841_-_8/1-50

GTTCTTAGAAGTTTTGGGGCTTTCGGCTGAACTAATCAGCGAAAATTAAG

>30_MC06_541993_544096_-_9/1-50

GTTCTTAGAAGTTCTGTGGTCTGGGGTTGGTTTGATCAGCGAAAATAAAC

>38_Rhigh_818886_821002_-_18/1-50

GTTCTTAGAAGTTTAGAGGTTTGGGACTCGTCTGATAGACGAAAATAAAC

>32_Rhigh_265343_267347_+_14/1-50

GATCTTAGAAGTTCTTAAGTTTGGGTCTCGTCTGATCGGTGAAAATTAAG

>42_NC08_772916_774962_-_11/1-50

---CTTAGAAGTTTAGAGGTTTGGGGCTCGTCTGATCGACGAAAATAAAC

>14_Rlow_595068_597117_-_11/1-50

GTTCTTAGAAGTTTAGGGGTATGGGGTTGGGCAAGTGAGCGAAAATAAAC

>46_NY01_800390_802466_-_4/1-50

GATTTTAGAAGTTCTTAAGTTTGGGCCCCGTCTGATCAGCGAAAATAAAC

>15_VA94_284363_286460_+_6/1-50

GTTCTTAGAAGTTAGGGGAGTTTGGTCTGGCTTGATCTGCGAAAATAAAC

>6_NY01_281167_283186_+_8/1-50

GTTCTTAGAAGTTTAGGAGTTAGCGGATGCTCTGATCAGCGAAAATAAAC

>25_Rhigh_838046_840170_-_6/1-50

GTTCTTAGAAGTTCAGGAGTGTCGGGTTGAGCTAGTGAGCGAAAATTAAG
